# Rational Sine-Gordon expansion method to analyze the dynamical behavior of the time-fractional phi-four and (2 + 1) dimensional CBS equations

**DOI:** 10.1038/s41598-024-60156-w

**Published:** 2024-04-24

**Authors:** Abdulla-Al- Mamun, Chunhui Lu, Samsun Nahar Ananna, Md Mohi Uddin

**Affiliations:** 1 College of Hydrology and Water Resources, Hohai University, Nanjing, 210098 People’s Republic of China; 2 State Key Laboratory of Hydrology-Water Resources and Hydraulic Engineering, Hohai University, Nanjing, People’s Republic of China; 3 Department of Computer Science and Engineering, Northern University of Business and Technology Khulna, Khulna, 9100 Bangladesh; 4 School of Mathematics, Hohai University, Nanjing, 210098 People’s Republic of China; 5 College of Water Conservancy and Hydropower Engineering, Hohai University, Nanjing, 210098 People’s Republic of China

**Keywords:** The rational sine-Gordon expansion (RSGE) method, Phi-four equation, Soliton wave, Travelling wave solution, Calogero-Bogoyavlanskil Schilf equation, Sine-Gordon, Water wave, Engineering, Mathematics and computing, Optics and photonics

## Abstract

This study uses the rational Sine-Gordon expansion (RSGE) method to investigate the dynamical behavior of traveling wave solutions of the water wave phenomena for the time-fractional phi-four equation and the (2 + 1) dimensional Calogero-Bogoyavlanskil schilf (CBS) equation based on the conformable derivative. The technique uses the sine-Gordon equation as an auxiliary equation to generalize the well-known sine-Gordon expansion. It adopts a more broad strategy, a rational function rather than a polynomial one, of the solutions of the auxiliary equation, in contrast to the traditional sine-Gordon expansion technique. Several explanations for hyperbolic functions may be produced using the previously stated approach. The approach mentioned above is employed to provide diverse solutions of the time-fractional phi-four equation and the (2 + 1) dimensional CBS equations involving hyperbolic functions, such as soliton, single soliton, multiple-soliton, kink, cusp, lump-kink, kink double-soliton, and others. The RSGE approach enhances our comprehension of nonlinear processes, offers precise solutions to nonlinear equations, facilitates the investigation of solitons, propels the development of mathematical tools, and is applicable in many scientific and technical fields. The solutions are graphically shown in three-dimensional (3D) surface and contour plots using MATLAB software. All screens display the absolute wave configurations in the resolutions of the equation with the proper parameters. Furthermore, it can be deduced that the physical properties of the found solutions and their characteristics may help us comprehend how shallow water waves move in nonlinear dynamics.

## Introduction

To characterize the physical properties of various applied science problems, such as fluid dynamics, hydrodynamics, plasma physics, and quantum mechanics, given the right circumstances, ordinary and partial differential equations may be used to represent the problems. Analytical solutions to partial differential equations (PDEs), particularly nonlinear equations, are more complex than those to ordinary differential equations (ODEs). PDEs frequently transform into ODEs using the Ansatz (direct) and Symmetry approaches to look for explicit solutions. Exact solutions help compare numerical systems and confirm accuracy. The endeavor to get precise, analytical solutions to PDEs is not just of an academic nature; it holds practical importance in verifying and comparing numerical and simulation techniques. In applied sciences, precise mathematical models and solutions are essential when doing direct experiments, which may be difficult or unfeasible. Although exact solutions are beneficial, they are infamously challenging for most nonlinear partial differential equations (PDEs), which are commonly used to represent real-world processes. The complexity derives from the inherent nonlinearity, which frequently gives rise to intricate phenomena like chaos, turbulence, and wave breaking, which lack straightforward analytical explanations. Despite the difficulties, current research in mathematical methods, computational techniques, and theoretical physics is still progressing and increasing the number of partial differential equations (PDEs) for which we have exact solutions or effective approximation methods. This improves our understanding and ability to model complex systems in applied sciences.

The (2 + 1)-dimensional CBS equation is a nonlinear partial differential equation and can exhibit various solutions, including solitons, rogue waves, and other nonlinear wave patterns. It has applications in studying multiple physical systems, including fluid dynamics, plasma physics, and nonlinear optics. Consider the subsequent generalized (2 + 1)-dimensional CBS circumstances:

1 $${u}_{t}+\phi \left(u\right){u}_{y}=0, \phi \left(u\right)={\partial }_{x}^{2}+au+b{u}_{x}{\partial }_{x}^{-1},$$

or homogeneously,

2 $${u}_{t}+{u}_{xxy}+au{u}_{y}+b{v}_{x}{\partial }_{x}^{-1}{v}_{y}=0,$$

where ${\partial }_{x}^{-1}=\int fdx$ and a, b are constraints. Equation (2) can be characterized in the probable time-fractional form of the CBS equation^1,2^^.^

3 $${u}_{x}{D}_{t}^{\theta }u+4{u}_{x}{u}_{xy}+2{u}_{xx}{u}_{y}+{u}_{xxxy}=0, t>0, x,y\in {\mathbb{R}},$$

where $$0<\theta \le 1$$.

The time-fractional Phi-Four equation is a partial differential equation that generalizes the standard Phi-Four equation by incorporating fractional derivatives concerning time. The standard Phi-Four equation is a well-known equation in mathematical physics, often used to describe certain phenomena in fields like condensed matter physics and nonlinear optics. Adding fractional derivatives in time allows for more complex behaviors that capture specific anomalous diffusion processes. Due to the inclusion of fractional derivatives, the behavior, and solutions of the time-fractional Phi-Four equation can be more intriguing and complex than those of the ordinary Phi-Four equation. Anomaly diffusion and other unusual behaviors may result from the non-locality and memory effect introduced by the fractional derivative. The Phi-four equation is a specific form of the Klein–Gordon equation^1^.

4 $${D}_{t}^{2\theta }u-{u}_{xx}+{\uplambda }^{2}u+\mu {u}^{3}=0, \gamma >0, 0<\theta \le 1$$

where $\uplambda$ and $\mu$ are real numbers.

The main goal of this work is to directly apply the RSGE method to the dynamical analysis of the time-fractional phi-four equation and the (2 + 1) dimensional CBS equation. There are several benefits when comparing our strategy to the other approaches. Simply put, it employs a more structured technique and more steps to generate an algebraic system. It also automatically creates kink and singular soliton solutions^3–5^. The principal important methodology of this method is too explicit the exact solutions of FNLEEs that satisfy the Nonlinear ODE of the form, $U\left(\uppsi \right)=\sum_{i=1}^{N}{{\text{tanh}}}^{i-1}\uppsi \left({a}_{i}\mathrm{sech\psi }+{c}_{i}\mathrm{tanh\psi }+{a}_{0}\right)/\sum_{i=1}^{N}{{\text{tanh}}}^{i-1}\uppsi \left({b}_{i}\mathrm{sech\psi }+{d}_{i}\mathrm{tanh\psi }+{b}_{0}\right)$. Our method provides a more direct and concise approach to the exact travelling wave solution than the other existing systems. Some authors used this RSGE technique to determine the exact solution to multiple NLEEs in the deferential sense of derivative, such as Jumarie's modified Riemann–Liouville derivatives, conformable derivatives, and Kerr law nonlinearity. Nevertheless, no adequate studies utilizing this method have been conducted on our suggested time-fractional phi-four equation and the (2 + 1) dimensional CBS equation. Here, the recently found exact solution of the time-fractional phi-four equation and the (2 + 1) dimensional CBS equations is more accurate, efficient, and versatile enough to be used in many treatments in mathematical physics, engineering, and wave analysis. Thus, we can state that our proposed research is innovative in the sense of conformable derivatives as it employs the RSGE technique to dynamically analyze the time-fractional phi-four equation and the (2 + 1) dimensional CBS equation. We presented the results using the mathematical software Mathematica by choosing appropriate values for the employed parameters and then employing illustrations to simplify the physical interpretation suitably.

To establish a flow in a domain, air must be replaced by water in soils (and foams), or vice versa, in fluid recovery activities. The principles governing fluid flow are the same in both systems. However, depending on the media, these regulations may be conveyed differently or utilize different languages. Although the scientific fields of flow in soil and flow in foam are concerned with similar physical laws^6–8^, communication between them has been impeded by a lack of common vocabulary. Water waves are a regular and fascinating example of traveling waves in nature. As a traveling wave passes over the water’s surface, the water’s surface oscillates up and down, creating wave patterns that move over the top. How water waves behave may be determined by their properties, including their wavelength, frequency, speed, and amplitude. The wave equation governs the dynamics of water waves, a partial differential equation that explains the relationship between wave motion, time, and space. The standard wave equation for small-amplitude waves in shallow water is the one-dimensional linear shallow water wave equation, sometimes referred to as the Korteweg-de Vries equation (KdV)^9^. It describes waves with a single wave profile and the ability to move without changing shape. In water, waves frequently disperse, which means they move at varied speeds depending on their wavelength. Longer waves with lower frequencies travel more quickly than shorter waves with higher frequencies.

**Figure 1:**
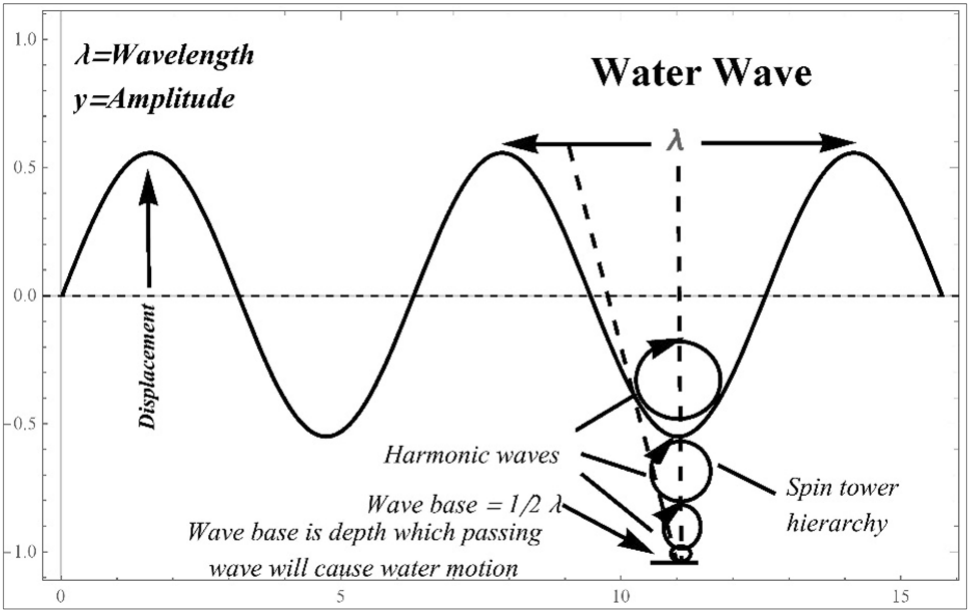
Dynamics of water waves.

This dispersion results from the waves' interactions with the surface tension, water depth, and waves. For waves with large amplitudes propagating over great distances, the dynamics of water waves can become nonlinear^10^. Rogue waves and solitons are intricate patterns that can develop due to nonlinear wave dynamics. Waves may get steeper and more unstable as they approach shallow water, eventually breaking into choppy whitecaps. This phenomenon is most noticeable near coastlines. Water waves, in general, show a wide variety of features, making them a significant subject of interest and research in fluid dynamics, oceanography, and other related fields. Wave characteristics, water depth, and interactions with the environment are only a few factors that influence how they behave dynamically^11,12^. Figure 1 shows the dynamics of water waves. The wavelength of a water wave, represented by the symbol $\lambda$, is the separation between two successive wave crests (or troughs). It symbolises the wave's spatial period, or the length of time the wave repeats its shape. A wave's wavelength in water is determined by a number of variables, such as the wave's frequency and depth. The connection between wavelength ($\lambda$), wave speed ($c$), and wave period ($T$) in deep water, when the depth is much higher than the wavelength, may be explained by $\lambda =c/f$. The amplitude of a water wave, represented by $y$. The amplitude of the water wave is measured vertically from the undisturbed water level (the equilibrium position) to the peak of the wave crest or the lowest point of the wave trough.

Nonlinear fractional differential equations have attracted a lot of attention lately. It significantly impacts how fractional calculus theory changes, and these forms are used in physics, engineering, and biology, among other domains^13^. Traveling wave solutions of nonlinear partial differential equations must be investigated to distinguish between various nonlinear situations in applied research and engineering. Only a few of the many nonlinear wave techniques that have been used in the past to illustrate various physics issues include heat flow, shallow water waves, wave propagation, optical fibers, plasma physics, fluid mechanics, biology, electricity, chemical kinematics, and quantum theory^14–18^. Thus, to investigate these substances, scores of effective strategies have been recommended in the circulated works by scholars, namely the improved modified extended tanh-function method^4^, the (G′/G,1/G)-expansion technique^19,20^, the modified extended tanh-function method^1,9,21^, the (${{\text{G}}}^{\mathrm{^{\prime}}}/{{\text{G}}}^{2}$)-expansion technique^22,23^, the advanced ${\text{exp}}\left(-\mathrm{\varnothing }\left(\upxi \right)\right)$-expansion method^24–26^, the tanh-coth method^27^, the variational iteration method^28–30^, the method of characteristics^31^, the multiple Exp-function method^32–34^, the sine-Gordon expansion method^3,35,36^, rational sine-Gordon expansion method^37,38^, Backlund transformations^39^, ultraspherical wavelets collocation method^40^, extended direct algebraic method^41^, the unified method^42^, the hyperbolic trigonometric method^43^, the new auxiliary equation method^5^, transformed rational function method^44^, the Hirota bilinear method^45–47^, the generalized Hirota bilinear method^48^, Soret and Dufour effects^49^, the rational ${\text{tan}}(K(\rho ))$-expansion technique^50^, the improved ${\text{tan}}(\Phi (\rho )/2)$-expansion technique^51^, the binary Hirota polynomial scheme^52^, the Kudryashov method^53^, etc.

The remainder of the paper is organized as follows. In Sect. “Algorithm of the RSGE method”, a basic description of the RSGE method is given. The mathematical formulation of the phi-four and CBS equations and their application using the RSGE approach are provided in Sect. “Application of the RSGE method”. Graphical depictions of the solutions discovered are given in Sect. “Result and Discussion”. The conclusions are presented in Sect. “Conclusion”.

## Algorithm of the RSGE method

The consistent fractional form $u\left(x,t\right)=U(\uppsi )$ with $\uppsi =a\left(x-\frac{v{t}^{\alpha }}{\alpha }\right)$ The unadventurous wave renovation^3,54–56^ decreases the fractional Sine-Gordon equation in one dimension of the form.

5 $$\frac{{\partial }^{2}u}{\partial {x}^{2}}-{D}_{t}^{2\alpha }u={m}^{2}{\text{sin}}u, m\, is\, constant.$$

To the ODE

6 $$\frac{{d}^{2}U}{d{\uppsi }^{2}}=\frac{{m}^{2}}{{a}^{2}\left(1-{v}^{2}\right)}{\text{sin}}U,$$

where $v$ indicates the velocity of the traveling wave illustrated in the transform^57^. Some simplifications lead

7 $${\left(\frac{d\left(\frac{U}{2}\right)}{d\uppsi }\right)}^{2}=\frac{{m}^{2}}{{a}^{2}\left(1-{v}^{2}\right)}{{\text{sin}}}^{2}\frac{U}{2}+C,$$

where C is an integrating constant and is supposed to be zero for simplicity. Let $w\left(\uppsi \right)=\frac{U\left(\uppsi \right)}{2}$ and ${b}^{2}=\frac{{m}^{2}}{{a}^{2}\left(1-{v}^{2}\right)}$. Then (7) is converted to

8 $$\frac{d\left(w\right)}{d\uppsi }=b{\text{sin}}w.$$

Set $b=1$ in (8). Then (8) yields two significant relations.

9 $${\text{sin}}w(\uppsi )={\left.\frac{2d{e}^{\uppsi }}{{d}^{2}{e}^{2\uppsi }+1}\right|}_{d=1}=\mathrm{sech\psi },$$

or

10 $${\text{cos}}w\left(\uppsi \right)={\left.\frac{{d}^{2}{e}^{2\uppsi }-1}{{d}^{2}{e}^{2\uppsi }+1}\right|}_{d=1}=\mathrm{tanh\psi },$$

where $d$ is a nonzero integrating constant. Then the fractional PDE of the form

11 $$P\left(u, {D}_{t}^{\alpha }u, {u}_{x}, {D}_{tt}^{2\alpha }, {u}_{xx}, \dots \dots \right)=0,$$

can be reduced to an ODE

12 $$\widetilde{P}=\left(U,{U}{\prime},{U}^{{\prime}{\prime}},\dots \dots \right)=0,$$

by using an equivalent wave transform $u\left(x,t\right)=U(\uppsi )$ where the transform variable $\uppsi$ is specified as $a\left(x-\frac{v{t}^{\alpha }}{\alpha }\right)$. Then, the expected solution (12) of the form

13 $$U\left(\uppsi \right)={A}_{0}+\sum_{i=1}^{s}{{\text{tanh}}}^{i-1}\left(\uppsi \right)\left({B}_{i}\,\mathrm{sech\, \psi }+{A}_{i}\,\mathrm{tanh\,\psi }\right),$$

can be written as

14 $$U\left(w\right)={A}_{0}+\sum_{i=1}^{s}{{\text{cos}}}^{i-1}\left(w\right)\left({B}_{i}\,{\text{sin}}\,w+{A}_{i}\,{\text{cos}}\,w\right).$$

use Eqs. (9) and (10), Eq. (13) is a bivariate polynomial function in $\mathrm{tanh\psi }$ and $\mathrm{sech\psi }$, as is evident. Due to the relationships ${{\text{tanh}}}^{2}\uppsi +{{\text{sech}}}^{2}\uppsi =1$, it is essential to note that this polynomial must be linear in one of these auxiliary functions. In this case, $\mathrm{sech\psi }$. We can now see that a subset of rational functions comprises polynomial functions. As a result, the latter is often far superior to the former in tasks like interpolation or approximating functions^58^. It is simple to assume that the same will hold while attempting to solve nonlinear evolution equations. The concept of rational expansion has been utilized in the literature before, but only in the context of one auxiliary function^58–60^. In this study, we propose expanding this concept to two additional tasks.

15 $$U\left(\uppsi \right)=\frac{\sum_{i=1}^{N}{{\text{tanh}}}^{i-1}\uppsi \left({a}_{i}\,\mathrm{sech\,\psi }+{c}_{i}\,\mathrm{tanh\,\psi }+{a}_{0}\right)}{\sum_{i=1}^{N}{{\text{tanh}}}^{i-1}\uppsi \left({b}_{i}\,\mathrm{sech\,\psi }+{d}_{i}\,\mathrm{tanh\,\psi }+{b}_{0}\right)},$$

in place of Eq. (13), which can also be written as

16 $$U\left({\text{w}}\right)=\frac{\sum_{i=1}^{N}{{\text{cos}}}^{i-1}{\text{w}}\left({a}_{i}\,{\text{sin\,w}}+{c}_{i}\,{\text{cos\,w}}+{a}_{0}\right)}{\sum_{i=1}^{N}{{\text{cos}}}^{i-1}{\text{w}}\left({b}_{i}\,{\text{sin\,w}}+{d}_{i}\,{\text{cos\,w}}+{b}_{0}\right)},$$

owing to (15)–(16). Setting up index limits with a uniform balance of the conditions in (12) is the first step in the procedure. The projected solution (15), engaging in (12), is replaced, and the coefficient of powers of sin and cos is assumed to be zero. Next, the coefficients are explained by the ensuing algebraic system. ${a}_{0},{a}_{1},{b}_{0},{b}_{1},\dots \dots$.. If there are any answers, they are put together using (9)–(10) and $\uppsi$.

## Application of the RSGE method

### Application for the Phi-four equation

Employing the subsequent traveling wave transformation

$${\text{u}}\left({\text{x}},{\text{t}}\right)={\text{U}}\left(\psi \right),\mathrm{where }\,\psi ={\text{q}}x-p\frac{{t}^{\theta }}{\theta }.$$

on Eq. (4), we get

17 $$\left({{\text{p}}}^{2}-{{\text{q}}}^{2}\right){{\text{U}}}^{\mathrm{^{\prime}}\mathrm{^{\prime}}}+{\uplambda }^{2}{\text{U}}+\upmu {{\text{U}}}^{3}=0,$$

With the asset of standardized balancing of the highest order derivative term ${U}^{\mathrm{^{\prime}}\mathrm{^{\prime}}}$ and nonlinear term ${U}^{3}$ in Eq. 17, we find that $N=1$. Therefore, the auxiliary solution becomes:

18 $$\begin{array}{l}\mu {a}_{0}^{3}+3\mu {a}_{0}{a}_{1}^{2}+{\lambda }^{2}{a}_{0}{b}_{0}^{2}-{p}^{2}{a}_{1}{b}_{0}{b}_{1}+{q}^{2}{a}_{1}{b}_{0}{b}_{1}+2{\lambda }^{2}{a}_{1}{b}_{0}{b}_{1}-{p}^{2}{a}_{0}{b}_{1}^{2}+{q}^{2}{a}_{0}{b}_{1}^{2}+{\lambda }^{2}{a}_{0}{b}_{1}^{2}\\ -2{p}^{2}{b}_{0}{c}_{1}{d}_{1}+2{q}^{2}{b}_{0}{c}_{1}{d}_{1}+2{p}^{2}{a}_{0}{d}_{1}^{2}-2{q}^{2}{a}_{0}{d}_{1}^{2}=0,\\ 3\mu {a}_{0}^{2}{c}_{1}+3\mu {a}_{1}^{2}{c}_{1}-2{p}^{2}{b}_{0}^{2}{c}_{1}+2{q}^{2}{b}_{0}^{2}{c}_{1}+{\lambda }^{2}{b}_{0}^{2}{c}_{1}+{p}^{2}{b}_{1}^{2}{c}_{1}-{q}^{2}{b}_{1}^{2}{c}_{1}+{\lambda }^{2}{b}_{1}^{2}{c}_{1}+2{p}^{2}{a}_{0}{b}_{0}{d}_{1}\\ -2{q}^{2}{a}_{0}{b}_{0}{d}_{1}+2{\lambda }^{2}{a}_{0}{b}_{0}{d}_{1}-{p}^{2}{a}_{1}{b}_{1}{d}_{1}+{q}^{2}{a}_{1}{b}_{1}{d}_{1}+2{\lambda }^{2}{a}_{1}{b}_{1}{d}_{1}=0,\\ -3\mu {a}_{0}{a}_{1}^{2}+{p}^{2}{a}_{1}{b}_{0}{b}_{1}-{q}^{2}{a}_{1}{b}_{0}{b}_{1}-2{\lambda }^{2}{a}_{1}{b}_{0}{b}_{1}+3{p}^{2}{a}_{0}{b}_{1}^{2}-3{q}^{2}{a}_{0}{b}_{1}^{2}-{\lambda }^{2}{a}_{0}{b}_{1}^{2}+3\mu {a}_{0}{c}_{1}^{2}\\ +2{p}^{2}{b}_{0}{c}_{1}{d}_{1}-2{q}^{2}{b}_{0}{c}_{1}{d}_{1}+2{\lambda }^{2}{b}_{0}{c}_{1}{d}_{1}-2{p}^{2}{a}_{0}{d}_{1}^{2}+2{q}^{2}{a}_{0}{d}_{1}^{2}+{\lambda }^{2}{a}_{0}{d}_{1}^{2}=0,\\ -3\mu {a}_{1}^{2}{c}_{1}+2{p}^{2}{b}_{0}^{2}{c}_{1}-2{q}^{2}{b}_{0}^{2}{c}_{1}-{p}^{2}{b}_{1}^{2}{c}_{1}+{q}^{2}{b}_{1}^{2}{c}_{1}-{\lambda }^{2}{b}_{1}^{2}{c}_{1}+\mu {c}_{1}^{3}-2{p}^{2}{a}_{0}{b}_{0}{d}_{1}+2{q}^{2}{a}_{0}{b}_{0}{d}_{1}\\ +{p}^{2}{a}_{1}{b}_{1}{d}_{1}-{q}^{2}{a}_{1}{b}_{1}{d}_{1}-2{\lambda }^{2}{a}_{1}{b}_{1}{d}_{1}+{\lambda }^{2}{c}_{1}{d}_{1}^{2}=0,\\ -2{p}^{2}{a}_{0}{b}_{1}^{2}+2{q}^{2}{a}_{0}{b}_{1}^{2}=0,\\ \mu {a}_{0}^{3}+3\mu {a}_{0}{a}_{1}^{2}+{\lambda }^{2}{a}_{0}{b}_{0}^{2}-{p}^{2}{a}_{1}{b}_{0}{b}_{1}+{q}^{2}{a}_{1}{b}_{0}{b}_{1}+2{\lambda }^{2}{a}_{1}{b}_{0}{b}_{1}-{p}^{2}{a}_{0}{b}_{1}^{2}+{q}^{2}{a}_{0}{b}_{1}^{2}+{\lambda }^{2}{a}_{0}{b}_{1}^{2}\\ -2{p}^{2}{b}_{0}{c}_{1}{d}_{1}+2{q}^{2}{b}_{0}{c}_{1}{d}_{1}+2{p}^{2}{a}_{0}{d}_{1}^{2}-2{q}^{2}{a}_{0}{d}_{1}^{2}=0,\\ \begin{array}{l}3\mu {a}_{0}^{2}{a}_{1}+\mu {a}_{1}^{3}-{p}^{2}{a}_{1}{b}_{0}^{2}+{q}^{2}{a}_{1}{b}_{0}^{2}+{\lambda }^{2}{a}_{1}{b}_{0}^{2}-{p}^{2}{a}_{0}{b}_{0}{b}_{1}+{q}^{2}{a}_{0}{b}_{0}{b}_{1}+2{\lambda }^{2}{a}_{0}{b}_{0}{b}_{1}+{\lambda }^{2}{a}_{1}{b}_{1}^{2}\\ -2{p}^{2}{b}_{1}{c}_{1}{d}_{1}+2{q}^{2}{b}_{1}{c}_{1}{d}_{1}+2{p}^{2}{a}_{1}{d}_{1}^{2}-2{q}^{2}{a}_{1}{d}_{1}^{2}=0,\\ 6\mu {a}_{0}{a}_{1}{c}_{1}-{p}^{2}{b}_{0}{b}_{1}{c}_{1}+{q}^{2}{b}_{0}{b}_{1}{c}_{1}+2{\lambda }^{2}{b}_{0}{b}_{1}{c}_{1}+2{p}^{2}{a}_{1}{b}_{0}{d}_{1}-2{q}^{2}{a}_{1}{b}_{0}{d}_{1}+2{\lambda }^{2}{a}_{1}{b}_{0}{d}_{1}\\ -3{p}^{2}{a}_{0}{b}_{1}{d}_{1}+3{q}^{2}{a}_{0}{b}_{1}{d}_{1}+2{\lambda }^{2}{a}_{0}{b}_{1}{d}_{1}=0,\\ -\mu {a}_{1}^{3}+2{p}^{2}{a}_{1}{b}_{0}^{2}-2{q}^{2}{a}_{1}{b}_{0}^{2}-{\lambda }^{2}{a}_{1}{b}_{1}^{2}+3\mu {a}_{1}{c}_{1}^{2}+{p}^{2}{b}_{1}{c}_{1}{d}_{1}-{q}^{2}{b}_{1}{c}_{1}{d}_{1}+2{\lambda }^{2}{b}_{1}{c}_{1}{d}_{1}\\ -{p}^{2}{a}_{1}{d}_{1}^{2}+{q}^{2}{a}_{1}{d}_{1}^{2}+{\lambda }^{2}{a}_{1}{d}_{1}^{2}=0,\\ 2{p}^{2}{a}_{0}{b}_{1}{d}_{1}-2{q}^{2}{a}_{0}{b}_{1}{d}_{1}=0.\end{array}\end{array}$$

Solving the SAE Eq. (18) for ${\text{q}}, {{\text{A}}}_{0},{{\text{A}}}_{1},{{\text{B}}}_{1}$ we get several solutions sets as follows:

$$q=-\sqrt{{p}^{2}-2{\lambda }^{2}}, {a}_{0}=0, {a}_{1}=\pm \frac{\sqrt{{\lambda }^{2}{b}_{0}^{2}-{\lambda }^{2}{b}_{1}^{2}}}{\sqrt{\mu }}, {c}_{1}=\pm \frac{i\lambda {b}_{0}}{\sqrt{\mu }}, {d}_{1}=0.$$

$$q=\sqrt{{p}^{2}-2{\lambda }^{2}}, {a}_{0}=0, {a}_{1}=\pm \frac{\sqrt{{\lambda }^{2}{b}_{0}^{2}-{\lambda }^{2}{b}_{1}^{2}}}{\sqrt{\mu }}, {c}_{1}=\pm \frac{i\lambda {b}_{0}}{\sqrt{\mu }}, {d}_{1}=0.$$

$$q=-\sqrt{{p}^{2}-2{\lambda }^{2}}, {a}_{0}=\pm \frac{i\lambda {d}_{1}}{\sqrt{\mu }}, {a}_{1}=\pm \frac{\sqrt{{\lambda }^{2}{b}_{0}^{2}-{\lambda }^{2}{d}_{1}^{2}}}{\sqrt{\mu }}, {b}_{1}=0, {c}_{1}=\pm \frac{i\lambda {b}_{0}}{\sqrt{\mu }}.$$

$$q=\sqrt{{p}^{2}-2{\lambda }^{2}}, {a}_{0}=\pm \frac{i\lambda {d}_{1}}{\sqrt{\mu }}, {a}_{1}=\pm \frac{\sqrt{{\lambda }^{2}{b}_{0}^{2}-{\lambda }^{2}{d}_{1}^{2}}}{\sqrt{\mu }}, {b}_{1}=0, {c}_{1}=\pm \frac{i\lambda {b}_{0}}{\sqrt{\mu }}.$$

$$q=-\frac{\sqrt{2{p}^{2}-{\lambda }^{2}}}{\sqrt{2}}, {a}_{0}=-\frac{i\lambda {d}_{1}}{\sqrt{\mu }}, {a}_{1}=0, {b}_{1}=0, {c}_{1}=-\frac{i\lambda {b}_{0}}{\sqrt{\mu }}.$$

$$q=-\frac{\sqrt{2{p}^{2}-{\lambda }^{2}}}{\sqrt{2}}, {a}_{0}=\frac{i\lambda {d}_{1}}{\sqrt{\mu }}, {a}_{1}=0, {b}_{1}=0, {c}_{1}=\frac{i\lambda {b}_{0}}{\sqrt{\mu }}.$$

$$q=\frac{\sqrt{2{p}^{2}-{\lambda }^{2}}}{\sqrt{2}}, {a}_{0}=-\frac{i\lambda {d}_{1}}{\sqrt{\mu }}, {a}_{1}=0, {b}_{1}=0, {c}_{1}=-\frac{i\lambda {b}_{0}}{\sqrt{\mu }}.$$

$$q=\frac{\sqrt{2{p}^{2}-{\lambda }^{2}}}{\sqrt{2}}, {a}_{0}=\frac{i\lambda {d}_{1}}{\sqrt{\mu }}, {a}_{1}=0, {b}_{1}=0, {c}_{1}=\frac{i\lambda {b}_{0}}{\sqrt{\mu }}.$$

$$q=-\sqrt{{p}^{2}+{\lambda }^{2}}, {a}_{0}=0, {a}_{1}=\pm \frac{\sqrt{2}\sqrt{-{\lambda }^{2}{b}_{0}^{2}+{\lambda }^{2}{d}_{1}^{2}}}{\sqrt{\mu }}, {b}_{1}=0, {c}_{1}=0.$$

$$q=\sqrt{{p}^{2}+{\lambda }^{2}}, {a}_{0}=0, {a}_{1}=\pm \frac{\sqrt{2}\sqrt{-{\lambda }^{2}{b}_{0}^{2}+{\lambda }^{2}{d}_{1}^{2}}}{\sqrt{\mu }}, {b}_{1}=0, {c}_{1}=0.$$

$$q=-\sqrt{{p}^{2}-2{\lambda }^{2}}, {a}_{0}=0, {a}_{1}=0, {b}_{0}=\pm {b}_{1}, {c}_{1}=\pm \frac{i\lambda {b}_{1}}{\sqrt{\mu }}, {d}_{1}=0.$$

$$q=\sqrt{{p}^{2}-2{\lambda }^{2}}, {a}_{0}=0, {a}_{1}=0, {b}_{0}=\pm {b}_{1}, {c}_{1}=\pm \frac{i\lambda {b}_{1}}{\sqrt{\mu }}, {d}_{1}=0.$$

$$q=-\sqrt{{p}^{2}-2{\lambda }^{2}}, {a}_{0}=\pm \frac{i\lambda {d}_{1}}{\sqrt{\mu }}, {a}_{1}=\pm \frac{i\lambda {d}_{1}}{\sqrt{\mu }}, {b}_{0}=0, {b}_{1}=0, {c}_{1}=0.$$

$$q=-\sqrt{{p}^{2}-2{\lambda }^{2}}, {a}_{0}=0, {a}_{1}=\pm \frac{\lambda {b}_{0}}{\sqrt{\mu }}, {b}_{1}=0, {c}_{1}=\pm \frac{i\lambda {b}_{0}}{\sqrt{\mu }}, {d}_{1}=0.$$

$$q=\sqrt{{p}^{2}-2{\lambda }^{2}}, {a}_{0}=\pm \frac{i\lambda {d}_{1}}{\sqrt{\mu }}, {a}_{1}=\pm \frac{i\lambda {d}_{1}}{\sqrt{\mu }}, {b}_{0}=0, {b}_{1}=0, {c}_{1}=0.$$

$$q=\sqrt{{p}^{2}-2{\lambda }^{2}}, {a}_{0}=0, {a}_{1}=\pm \frac{\lambda {b}_{0}}{\sqrt{\mu }}, {b}_{1}=0, {c}_{1}=\pm \frac{i\lambda {b}_{0}}{\sqrt{\mu }}, {d}_{1}=0.$$

$$q=\pm \frac{\sqrt{2{p}^{2}-{\lambda }^{2}}}{\sqrt{2}}, {a}_{0}=0, {a}_{1}=0, {b}_{1}=0, {c}_{1}=\pm \frac{i\lambda {b}_{0}}{\sqrt{\mu }}, {d}_{1}=0.$$

$$q=-\sqrt{{p}^{2}+{\lambda }^{2}}, {a}_{0}=0, {a}_{1}=\pm \frac{\sqrt{2}\lambda {d}_{1}}{\sqrt{\mu }}, {b}_{0}=0, {b}_{1}=0, {c}_{1}=0.$$

$$q=-\sqrt{{p}^{2}+{\lambda }^{2}}, {a}_{0}=0, {a}_{1}=\pm \frac{i\lambda {d}_{1}}{\sqrt{\mu }}, {b}_{0}=\pm \sqrt{\frac{3}{2}} {d}_{1}, {b}_{1}=0, {c}_{1}=0.$$

$$q=-\sqrt{{p}^{2}+{\lambda }^{2}}, {a}_{0}=0, {a}_{1}=\pm \frac{i\sqrt{2}\lambda {d}_{1}}{\sqrt{\mu }}, {b}_{0}=-\sqrt{2}{d}_{1}, {b}_{1}=0, {c}_{1}=0.$$

$$q=-\sqrt{{p}^{2}+{\lambda }^{2}}, {a}_{0}=0, {a}_{1}=\pm \frac{i\sqrt{2}\lambda {d}_{1}}{\sqrt{\mu }}, {b}_{0}=\sqrt{2}{d}_{1}, {b}_{1}=0, {c}_{1}=0.$$

$$q=-\sqrt{{p}^{2}+{\lambda }^{2}}, {a}_{0}=0, {a}_{1}=\pm \frac{i\sqrt{2}\lambda {b}_{0}}{\sqrt{\mu }}, {b}_{1}=0, {c}_{1}=0, {d}_{1}=0.$$

$$q=\sqrt{{p}^{2}+{\lambda }^{2}}, {a}_{0}=0, {a}_{1}=\pm \frac{\sqrt{2}\lambda {d}_{1}}{\sqrt{\mu }}, {b}_{0}=0, {b}_{1}=0, {c}_{1}=0.$$

$$q=\sqrt{{p}^{2}+{\lambda }^{2}}, {a}_{0}=0, {a}_{1}=\pm \frac{i\lambda {d}_{1}}{\sqrt{\mu }}, {b}_{0}=\pm \sqrt{\frac{3}{2}} {d}_{1}, {b}_{1}=0, {c}_{1}=0.$$

$$q=\sqrt{{p}^{2}+{\lambda }^{2}}, {a}_{0}=0, {a}_{1}=\pm \frac{i\sqrt{2}\lambda {d}_{1}}{\sqrt{\mu }}, {b}_{0}=\pm \sqrt{2}{d}_{1}, {b}_{1}=0, {c}_{1}=0.$$

$$q=\sqrt{{p}^{2}+{\lambda }^{2}}, {a}_{0}=0, {a}_{1}=\pm \frac{i\sqrt{2}\lambda {b}_{0}}{\sqrt{\mu }}, {b}_{1}=0, {c}_{1}=0, {d}_{1}=0.$$

We obtained the precise Eq. (4) solutions concerning these solution sets.

$${U}_{\mathrm{1,2},\mathrm{3,4}}\left(x,t\right)=\pm \frac{i\lambda {\text{Sinh}}\left[\psi \right]{b}_{0}\pm \sqrt{{\lambda }^{2}\left({b}_{0}^{2}-{b}_{1}^{2}\right)}}{\sqrt{\mu }\left({\text{Cosh}}\left[\psi \right]{b}_{0}+{b}_{1}\right)}; \psi =-\frac{p{t}^{\theta }}{\theta }-x\sqrt{{p}^{2}-2{\lambda }^{2}}.$$

$${U}_{\mathrm{5,6},\mathrm{7,8}}\left(x,t\right)=\pm \frac{i\lambda {\text{Sinh}}\left[\psi \right]{b}_{0}\pm \sqrt{{\lambda }^{2}\left({b}_{0}^{2}-{b}_{1}^{2}\right)}}{\sqrt{\mu }\left({\text{Cosh}}\left[\psi \right]{b}_{0}+{b}_{1}\right)}; \psi =-\frac{p{t}^{\theta }}{\theta }+x\sqrt{{p}^{2}-2{\lambda }^{2}}.$$

$${U}_{\mathrm{9,10,11,12}}\left(x,t\right)=\pm \frac{i\lambda {d}_{1}\pm {\text{Sech}}[\psi ]\sqrt{{\lambda }^{2}({b}_{0}^{2}-{d}_{1}^{2})}+i\lambda {b}_{0}{\text{Tanh}}[\psi ]}{\sqrt{\mu }({b}_{0}+{d}_{1}{\text{Tanh}}[\psi ])}; \psi =-\frac{p{t}^{\theta }}{\theta }-x\sqrt{{p}^{2}-2{\lambda }^{2}}.$$

$${U}_{\mathrm{13,14,15,16}}\left(x,t\right)=\pm \frac{i\lambda {d}_{1}\pm {\text{Sech}}[\psi ]\sqrt{{\lambda }^{2}({b}_{0}^{2}-{d}_{1}^{2})}+i\lambda {b}_{0}{\text{Tanh}}[\psi ]}{\sqrt{\mu }({b}_{0}+{d}_{1}{\text{Tanh}}[\psi ])}; \psi =-\frac{p{t}^{\theta }}{\theta }+x\sqrt{{p}^{2}-2{\lambda }^{2}}.$$

$${U}_{\mathrm{17,18}}\left(x,t\right)=\pm \frac{i\lambda \left({d}_{1}+{b}_{0}{\text{Tanh}}\left[\psi \right]\right)}{\sqrt{\mu }({b}_{0}+{d}_{1}{\text{Tanh}}[\psi ])}; \psi =-\frac{p{t}^{\theta }}{\theta }-\frac{x\sqrt{2{p}^{2}-{\lambda }^{2}}}{\sqrt{2}}.$$

$${U}_{\mathrm{19,20}}\left(x,t\right)=\pm \frac{\sqrt{2}{\text{Sech}}[\psi ]\sqrt{-{\lambda }^{2}\left({b}_{0}^{2}-{d}_{1}^{2}\right)}}{\sqrt{\mu }({b}_{0}+{d}_{1}{\text{Tanh}}[\psi ])}; \psi =-\frac{p{t}^{\theta }}{\theta }-x\sqrt{{p}^{2}+{\lambda }^{2}}.$$

$${U}_{\mathrm{21,22}}\left(x,t\right)=\pm \frac{\sqrt{2}{\text{Sech}}[\psi ]\sqrt{-{\lambda }^{2}\left({b}_{0}^{2}-{d}_{1}^{2}\right)}}{\sqrt{\mu }({b}_{0}+{d}_{1}{\text{Tanh}}[\psi ])}; \psi =-\frac{p{t}^{\theta }}{\theta }+x\sqrt{{p}^{2}+{\lambda }^{2}}.$$

$${U}_{\mathrm{23,24}}\left(x,t\right)=\pm \frac{i\lambda {\text{Coth}}\left[\frac{\psi }{2}\right]}{\sqrt{\mu }}; \psi =-\frac{p{t}^{\theta }}{\theta }-x\sqrt{{p}^{2}-2{\lambda }^{2}}.$$

$${U}_{\mathrm{25,26}}\left(x,t\right)=\pm \frac{i\lambda {\text{Tanh}}\left[\frac{\psi }{2}\right]}{\sqrt{\mu }}; \psi =-\frac{p{t}^{\theta }}{\theta }-x\sqrt{{p}^{2}-2{\lambda }^{2}}.$$

$${U}_{\mathrm{27,28}}\left(x,t\right)=\pm \frac{i\lambda {\text{Coth}}\left[\frac{\psi }{2}\right]}{\sqrt{\mu }}; \psi =-\frac{p{t}^{\theta }}{\theta }+x\sqrt{{p}^{2}-2{\lambda }^{2}}.$$

$${U}_{\mathrm{29,30}}\left(x,t\right)=\pm \frac{i\lambda {\text{Tanh}}\left[\frac{\psi }{2}\right]}{\sqrt{\mu }}; \psi =-\frac{p{t}^{\theta }}{\theta }+x\sqrt{{p}^{2}-2{\lambda }^{2}}.$$

$${U}_{\mathrm{31,32,33,34}}\left(x,t\right)=\pm \frac{\lambda \left({\text{Sech}}\left[\psi \right]\pm i{\text{Tanh}}\left[\psi \right]\right)}{\sqrt{\mu }}; \psi =-\frac{p{t}^{\theta }}{\theta }-x\sqrt{{p}^{2}-2{\lambda }^{2}}.$$

$${U}_{\mathrm{35,36}}\left(x,t\right)=\pm \frac{i\lambda {\text{Tanh}}[\psi ]}{\sqrt{\mu }}; \psi =-\frac{p{t}^{\theta }}{\theta }-\frac{x\sqrt{2{p}^{2}-{\lambda }^{2}}}{\sqrt{2}}.$$

$${U}_{\mathrm{37,38}}\left(x,t\right)=\pm \frac{i\lambda {\text{Tanh}}[\psi ]}{\sqrt{\mu }}; \psi =-\frac{p{t}^{\theta }}{\theta }+\frac{x\sqrt{2{p}^{2}-{\lambda }^{2}}}{\sqrt{2}}.$$

$${U}_{\mathrm{39,40}}\left(x,t\right)=\pm \frac{\sqrt{2}\lambda {\text{Csch}}[\psi ]}{\sqrt{\mu }}; \psi =-\frac{p{t}^{\theta }}{\theta }-x\sqrt{{p}^{2}+{\lambda }^{2}}.$$

$${U}_{\mathrm{41,42,43,44}}\left(x,t\right)=\pm \frac{2i\lambda {\text{Sech}}[\psi ]}{\sqrt{\mu }(\sqrt{6}\pm 2{\text{Tanh}}[\psi ])}; \psi =-\frac{p{t}^{\theta }}{\theta }-x\sqrt{{p}^{2}+{\lambda }^{2}}.$$

$${U}_{\mathrm{45,46,47,48}}\left(x,t\right)=\pm \frac{2i\lambda {\text{Sech}}[\psi ]}{\sqrt{\mu }(\sqrt{6}\pm 2{\text{Tanh}}[\psi ])}; \psi =-\frac{p{t}^{\theta }}{\theta }+x\sqrt{{p}^{2}+{\lambda }^{2}}.$$

$${U}_{\mathrm{49,50}}\left(x,t\right)=\pm \frac{i\sqrt{2}\lambda {\text{Sech}}[\psi ]}{\sqrt{\mu }(\sqrt{2}-{\text{Tanh}}[\psi ])}; \psi =-\frac{p{t}^{\theta }}{\theta }-x\sqrt{{p}^{2}+{\lambda }^{2}}.$$

$${U}_{\mathrm{51,52}}\left(x,t\right)=\pm \frac{i\sqrt{2}\lambda {\text{Sech}}[\psi ]}{\sqrt{\mu }(\sqrt{2}+{\text{Tanh}}[\psi ])}; \psi =-\frac{p{t}^{\theta }}{\theta }+x\sqrt{{p}^{2}+{\lambda }^{2}}.$$

$${U}_{\mathrm{53,54}}\left(x,t\right)=\pm \frac{i\sqrt{2}\lambda {\text{Sech}}[\psi ]}{\sqrt{\mu }(\sqrt{2}+{\text{Tanh}}[\psi ])}; \psi =-\frac{p{t}^{\theta }}{\theta }-x\sqrt{{p}^{2}+{\lambda }^{2}}.$$

$${U}_{\mathrm{55,56}}\left(x,t\right)=\pm \frac{i\sqrt{2}\lambda {\text{Sech}}[\psi ]}{\sqrt{\mu }}; \psi =-\frac{p{t}^{\theta }}{\theta }-x\sqrt{{p}^{2}+{\lambda }^{2}}.$$

$${U}_{\mathrm{57,58}}\left(x,t\right)=\pm \frac{i\sqrt{2}\lambda {\text{Sech}}[\psi ]}{\sqrt{\mu }}; \psi =-\frac{p{t}^{\theta }}{\theta }+x\sqrt{{p}^{2}+{\lambda }^{2}}.$$

$${U}_{\mathrm{59,60}}\left(x,t\right)=\pm \frac{\sqrt{2}\lambda {\text{Csch}}[\psi ]}{\sqrt{\mu }}; \psi =-\frac{p{t}^{\theta }}{\theta }+x\sqrt{{p}^{2}+{\lambda }^{2}}.$$

### Application for the (2 + 1)-dimensional CBS equation

Employing the subsequent traveling wave transformation

19 $${\text{u}}\left({\text{x}},{\text{y}},{\text{t}}\right)={\text{U}}\left(\psi \right)\mathrm{ and }\psi ={\text{x}}+{\text{y}}-{\text{p}}\frac{{{\text{t}}}^{\uptheta }}{\uptheta },$$

on Eq. (3), we get,

20 $${{\text{pU}}}^{\mathrm{^{\prime}}}+\left(\frac{\uplambda +\upmu }{2}\right){\left({{\text{U}}}^{\mathrm{^{\prime}}}\right)}^{2}+{{\text{U}}}^{\mathrm{^{\prime}}\mathrm{^{\prime}}\mathrm{^{\prime}}}=0,$$

With the asset of standardized balancing of the highest order derivative term $U{\prime}{\prime}{\prime}$ and nonlinear term ${\left({U}{\prime}\right)}^{2}$ in Eq. 20, we find that $N=1$. Therefore, the auxiliary solution becomes:

$$p=-1, {a}_{1}=\frac{{a}_{0}{b}_{1}}{{b}_{0}}+\frac{6{b}_{1}{d}_{1}}{\left(\lambda +\mu \right){b}_{0}}\pm \frac{6\sqrt{{\left(\lambda +\mu \right)}^{2}{d}_{1}^{4}\left(-{b}_{0}^{2}+{b}_{1}^{2}+{d}_{1}^{2}\right)}}{{\left(\lambda +\mu \right)}^{2}{d}_{1}^{2}}, {c}_{1}=\frac{-6{b}_{0}^{2}+\lambda {a}_{0}{d}_{1}+\mu {a}_{0}{d}_{1}+6{d}_{1}^{2}}{\left(\lambda +\mu \right){b}_{0}}.$$

$$p=-1, {a}_{0}=-\frac{6{d}_{1}}{\lambda +\mu }, {a}_{1}=\frac{{\left(\lambda +\mu \right)}^{2}{b}_{1}{c}_{1}{d}_{1}\pm 6\sqrt{{\left(\lambda +\mu \right)}^{2}{d}_{1}^{4}\left({b}_{1}^{2}+{d}_{1}^{2}\right)}}{{\left(\lambda +\mu \right)}^{2}{d}_{1}^{2}}, {b}_{0}=0.$$

$$p=-1, {a}_{1}=\pm \frac{6\sqrt{-{b}_{0}^{2}+{d}_{1}^{2}}}{\sqrt{{\lambda }^{2}+2\lambda \mu +{\mu }^{2}}}, {b}_{1}=0, {c}_{1}=\frac{-6{b}_{0}^{2}+\lambda {a}_{0}{d}_{1}+\mu {a}_{0}{d}_{1}+6{d}_{1}^{2}}{\left(\lambda +\mu \right){b}_{0}}.$$

$$p=-4, {a}_{1}=0, {b}_{1}=0, {c}_{1}=\frac{-12{b}_{0}^{2}+\lambda {a}_{0}{d}_{1}+\mu {a}_{0}{d}_{1}+12{d}_{1}^{2}}{\left(\lambda +\mu \right){b}_{0}}.$$

$$p=-1, {a}_{1}=\frac{{\left(\lambda +\mu \right)}^{2}{a}_{0}{b}_{0}{b}_{1}\pm 6\sqrt{-{\left(\lambda +\mu \right)}^{2}{b}_{0}^{4}\left({b}_{0}^{2}-{b}_{1}^{2}\right)}}{{\left(\lambda +\mu \right)}^{2}{b}_{0}^{2}}, {c}_{1}=-\frac{6{b}_{0}}{\lambda +\mu }, {d}_{1}=0.$$

$$p=-1, {a}_{1}=\frac{\left(\lambda {a}_{0}+\mu {a}_{0}-12{b}_{0}\right){b}_{1}}{\left(\lambda +\mu \right){b}_{0}}, {c}_{1}=-{a}_{0}, {d}_{1}=-{b}_{0}.$$

$$p=-1, {a}_{1}=\frac{\left(\lambda {a}_{0}+\mu {a}_{0}+12{b}_{0}\right){b}_{1}}{\left(\lambda +\mu \right){b}_{0}}, {c}_{1}={a}_{0}, {d}_{1}={b}_{0}.$$

$$p=-1, {a}_{0}=-\frac{6{d}_{1}}{\lambda +\mu }, {a}_{1}=\pm \frac{6{d}_{1}}{\sqrt{{\lambda }^{2}+2\lambda \mu +{\mu }^{2}}}, {b}_{0}=0, {b}_{1}=0.$$

$$p=-4, {a}_{1}=0, {b}_{1}=0, {c}_{1}=-\frac{12{b}_{0}}{\lambda +\mu }, {d}_{1}=0.$$

$$p=-1, {a}_{1}=-\frac{6{b}_{0}}{\sqrt{-{\lambda }^{2}-2\lambda \mu -{\mu }^{2}}}, {b}_{1}=0, {c}_{1}=-\frac{6{b}_{0}}{\lambda +\mu }, {d}_{1}=0.$$

$$p=-1, {a}_{1}=\frac{6{b}_{0}}{\sqrt{-{\lambda }^{2}-2\lambda \mu -{\mu }^{2}}}, {b}_{1}=0, {c}_{1}=\frac{6{b}_{0}}{\lambda +\mu }, {d}_{1}=0.$$

We obtained the precise Eq. (3) solutions concerning these solution sets.

$${U}_{\mathrm{61,62}}\left(x,y,t\right)=\frac{{a}_{0}}{{b}_{0}}-\frac{6\left(\left(\lambda +\mu \right){\text{Sinh}}\left[\psi \right]{b}_{0}^{2}{d}_{1}^{2}-\left(\lambda +\mu \right){d}_{1}^{3}\left({b}_{1}+{\text{Sinh}}\left[\psi \right]{d}_{1}\right)\pm {b}_{0}\sqrt{{\left(\lambda +\mu \right)}^{2}{d}_{1}^{4}\left(-{b}_{0}^{2}+{b}_{1}^{2}+{d}_{1}^{2}\right)}\right)}{{\left(\lambda +\mu \right)}^{2}{b}_{0}{d}_{1}^{2}\left({\text{Cosh}}\left[\psi \right]{b}_{0}+{b}_{1}+{\text{Sinh}}\left[\psi \right]{d}_{1}\right)}; \psi =x+y+\frac{{t}^{\theta }}{\theta }.$$

$${U}_{\mathrm{63,64}}\left(x,y,t\right)=\frac{-6\left(\lambda +\mu \right){\text{Cosh}}\left[\psi \right]{d}_{1}^{3}+{\left(\lambda +\mu \right)}^{2}{c}_{1}{d}_{1}\left({b}_{1}+{\text{Sinh}}\left[\psi \right]{d}_{1}\right)\pm 6\sqrt{{\left(\lambda +\mu \right)}^{2}{d}_{1}^{4}\left({b}_{1}^{2}+{d}_{1}^{2}\right) }}{{\left(\lambda +\mu \right)}^{2}{d}_{1}^{2}\left({b}_{1}+{\text{Sinh}}\left[\psi \right]{d}_{1}\right)}; \psi =x+y+\frac{{t}^{\theta }}{\theta }.$$

$${U}_{\mathrm{65,66}}\left(x,y,t\right)=\frac{{a}_{0}\pm \frac{6{\text{Sech}}[\psi ]\sqrt{-{b}_{0}^{2}+{d}_{1}^{2}}}{\sqrt{{(\lambda +\mu )}^{2}}}+\frac{\left(-6{b}_{0}^{2}+{d}_{1}\left(\left(\lambda +\mu \right){a}_{0}+6{d}_{1}\right)\right){\text{Tanh}}\left[\psi \right]}{(\lambda +\mu ){b}_{0}}}{{b}_{0}+{d}_{1}{\text{Tanh}}[\psi ]}; \psi =x+y+\frac{{t}^{\theta }}{\theta }.$$

$${U}_{67}\left(x,y,t\right)=\frac{{a}_{0}+\frac{12\left(-{b}_{0}^{2}+{d}_{1}^{2}\right){\text{Tanh}}\left[\psi \right]}{(\lambda +\mu )({b}_{0}+{d}_{1}{\text{Tanh}}[\psi ])}}{{b}_{0}}; \psi =x+y+\frac{4{t}^{\theta }}{\theta }.$$

$${U}_{68}\left(x,y,t\right)=\frac{{a}_{0}{b}_{0}-\frac{6{\text{Sech}}\left[\psi \right]\left(\left(\lambda +\mu \right){\text{Sinh}}\left[\psi \right]{b}_{0}^{3}+\sqrt{-{\left(\lambda +\mu \right)}^{2}{b}_{0}^{4}\left({b}_{0}^{2}-{b}_{1}^{2}\right)}\right)}{{(\lambda +\mu )}^{2}({b}_{0}+{\text{Sech}}[\psi ]{b}_{1})}}{{b}_{0}^{2}}; \psi =x+y+\frac{{t}^{\theta }}{\theta }.$$

$${U}_{69}\left(x,y,t\right)=\frac{-6(\lambda +\mu ){\text{Sinh}}[\psi ]{b}_{0}^{3}+{(\lambda +\mu )}^{2}{a}_{0}{b}_{0}({\text{Cosh}}[\psi ]{b}_{0}+{b}_{1})+6\sqrt{-{(\lambda +\mu )}^{2}{b}_{0}^{4}({b}_{0}^{2}-{b}_{1}^{2})}}{{(\lambda +\mu )}^{2}{b}_{0}^{2}({\text{Cosh}}[\psi ]{b}_{0}+{b}_{1})}; \psi =x+y+\frac{{t}^{\theta }}{\theta }.$$

$${U}_{70}\left(x,y,t\right)=\frac{{a}_{0}}{{b}_{0}}-\frac{12{\text{Sech}}[\psi ]{b}_{1}}{(\lambda +\mu )({\text{Sech}}[\psi ]{b}_{1}-{b}_{0}(-1+{\text{Tanh}}[\psi ]))}; \psi =x+y+\frac{{t}^{\theta }}{\theta }.$$

$${U}_{71}\left(x,y,t\right)=\frac{{a}_{0}}{{b}_{0}}+\frac{12{b}_{1}}{(\lambda +\mu )({e}^{\psi }{b}_{0}+{b}_{1})}; \psi =x+y+\frac{{t}^{\theta }}{\theta }.$$

$${U}_{\mathrm{72,73}}\left(x,y,t\right)=-\frac{6{\text{Coth}}\left[\psi \right]}{\lambda +\mu }\pm \frac{6{\text{Csch}}[\psi ]}{\sqrt{{(\lambda +\mu )}^{2}}}+\frac{{c}_{1}}{{d}_{1}}; \psi =x+y+\frac{{t}^{\theta }}{\theta }.$$

$${U}_{74}\left(x,y,t\right)=\frac{{a}_{0}}{{b}_{0}}-\frac{12{\text{Tanh}}[\psi ]}{\lambda +\mu }; \psi =x+y+\frac{4{t}^{\theta }}{\theta }.$$

$${U}_{\mathrm{75,76}}\left(x,y,t\right)=\frac{6{\text{Sech}}[\psi ]}{\sqrt{-{(\lambda +\mu )}^{2}}}+\frac{{a}_{0}}{{b}_{0}}\pm \frac{6{\text{Tanh}}[\psi ]}{\lambda +\mu }; \psi =x+y+\frac{{t}^{\theta }}{\theta }.$$

## Result and discussion

**Figure 2:**
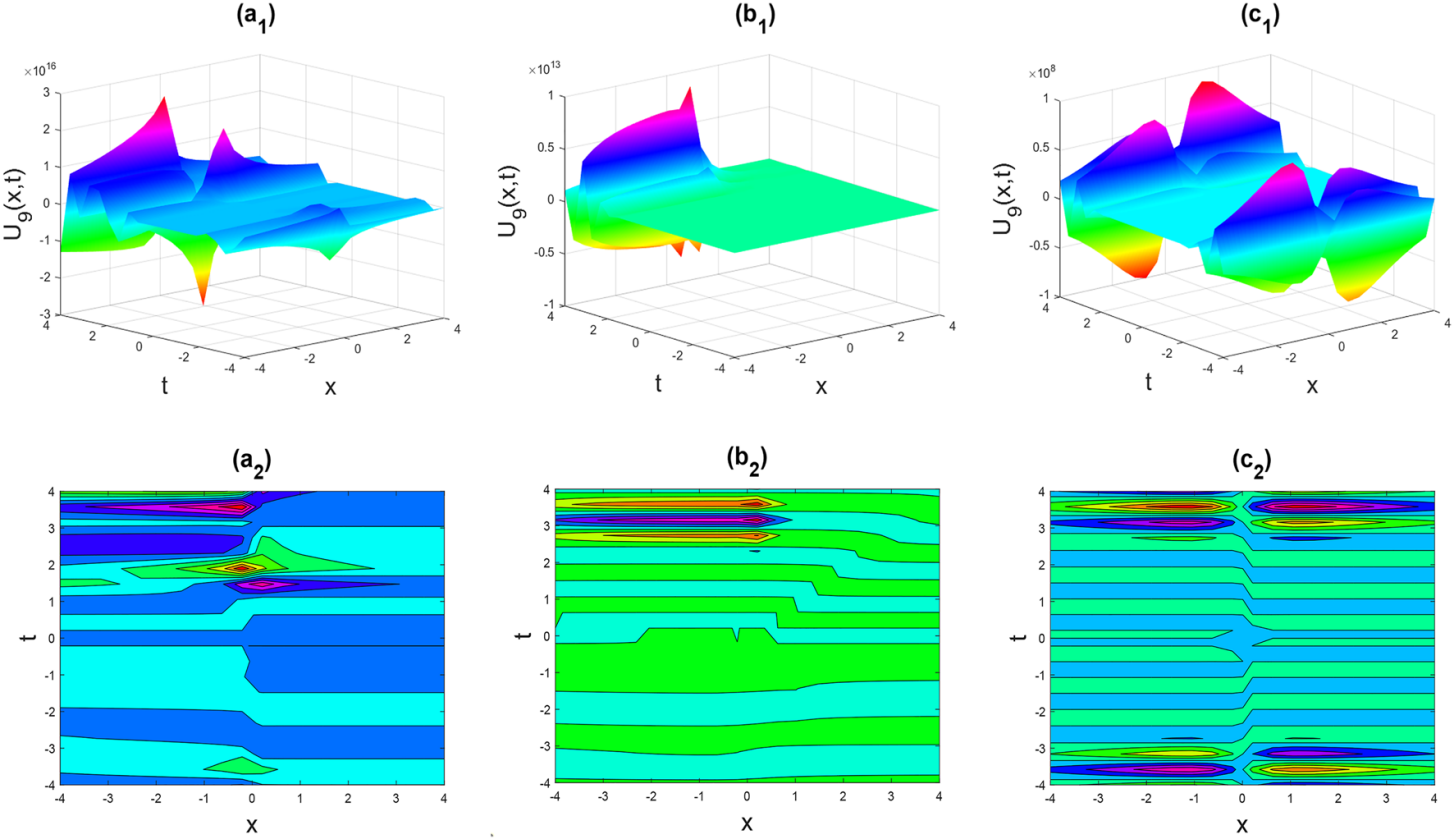
Kink-soliton shape of the imaginary part of ${U}_{9}\left(x,t\right)$ for the parameters $p=0.5, {b}_{0}=1, {d}_{1}=0.1, \lambda =1, \mu =1, \theta =0.3, 0.6, 1$.

**Figure 3:**
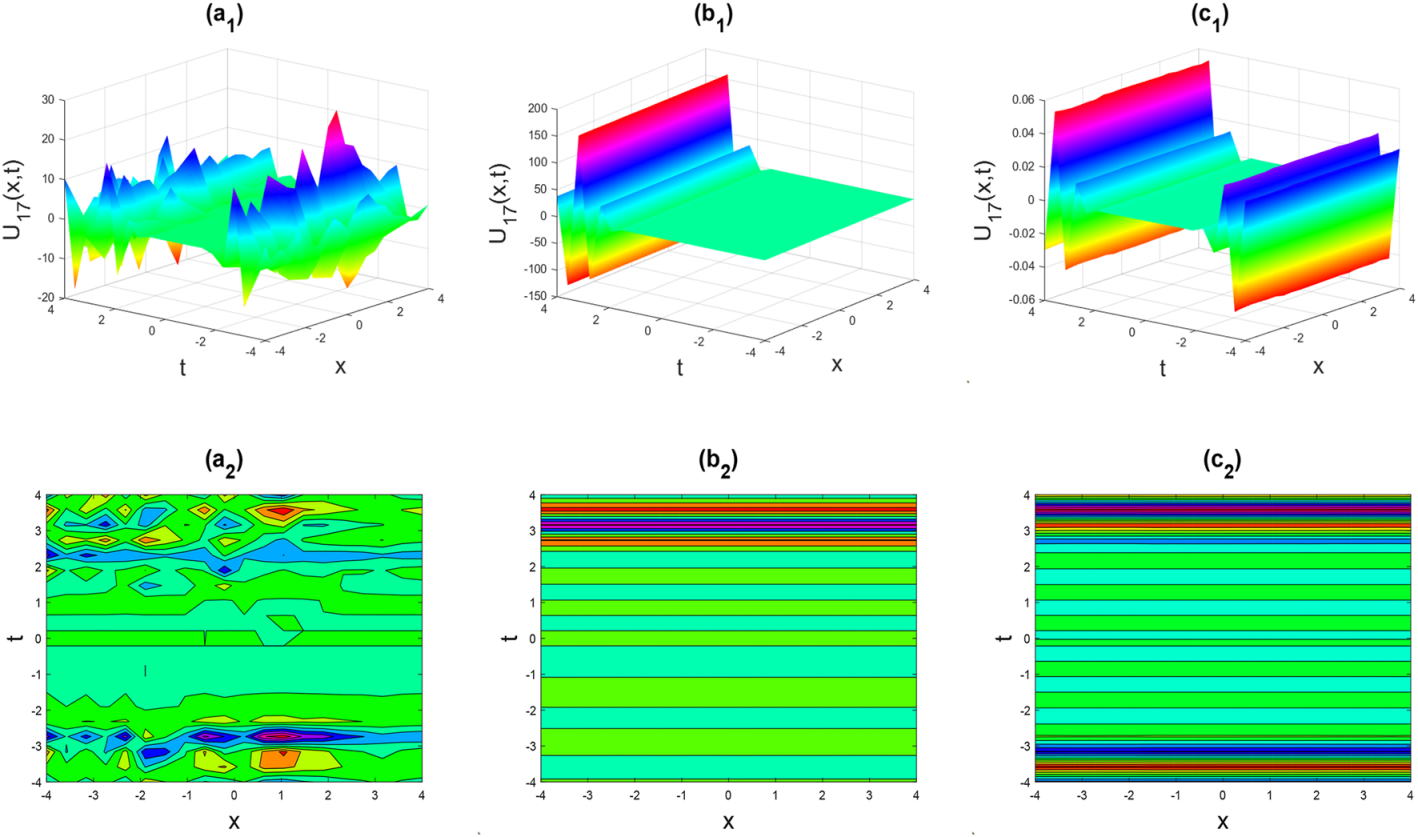
Kink-soliton shape of the real part of ${U}_{17}\left(x,t\right)$ for the parameters $p=0.5, {b}_{0}=1, {d}_{1}=0.1, \lambda =1, \mu =1, \theta =0.3, 0.6, 1$.

**Figure 4:**
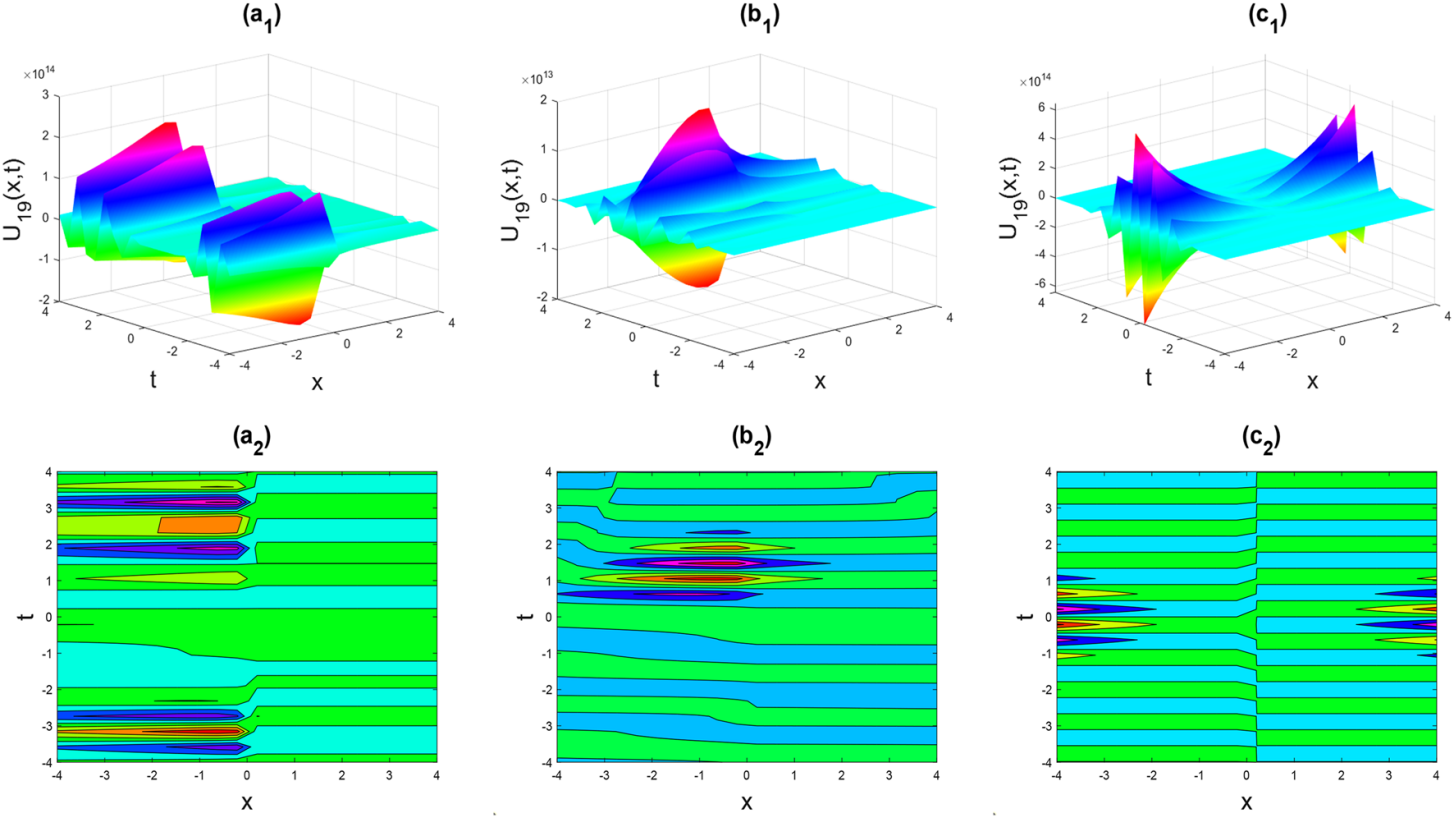
Kink shape of the imaginary part of ${U}_{19}\left(x,t\right)$ for the parameters $p=0.5, {b}_{0}=1, {d}_{1}=0.1, \lambda =1, \mu =1, \theta =0.3, 0.6, 1$.

**Figure 5:**
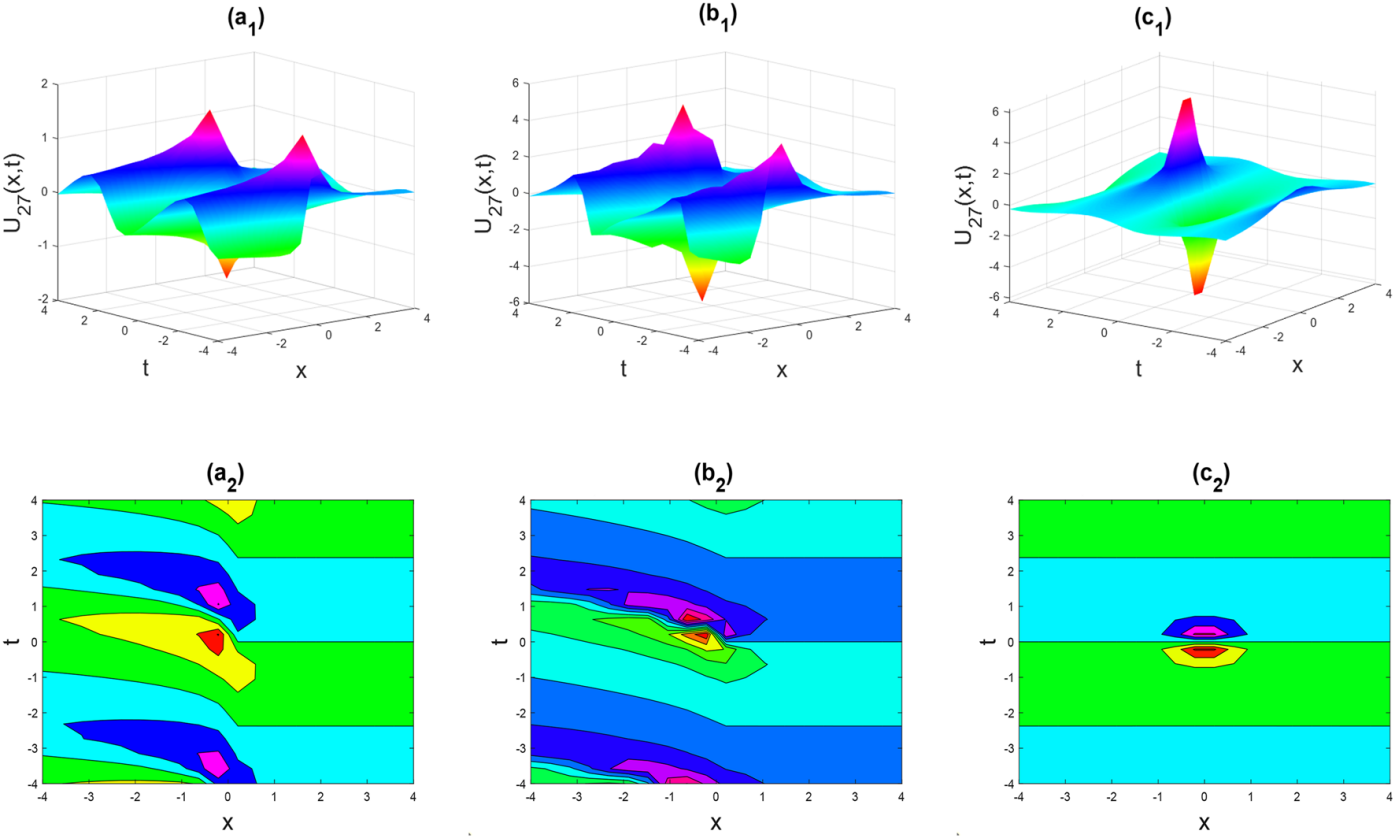
The rogue wave shape of the real part of ${U}_{27}\left(x,t\right)$ for the parameters $p=0.5, \lambda =1, \mu =1, \theta =0.3, 0.6, 1$.

**Figure 6:**
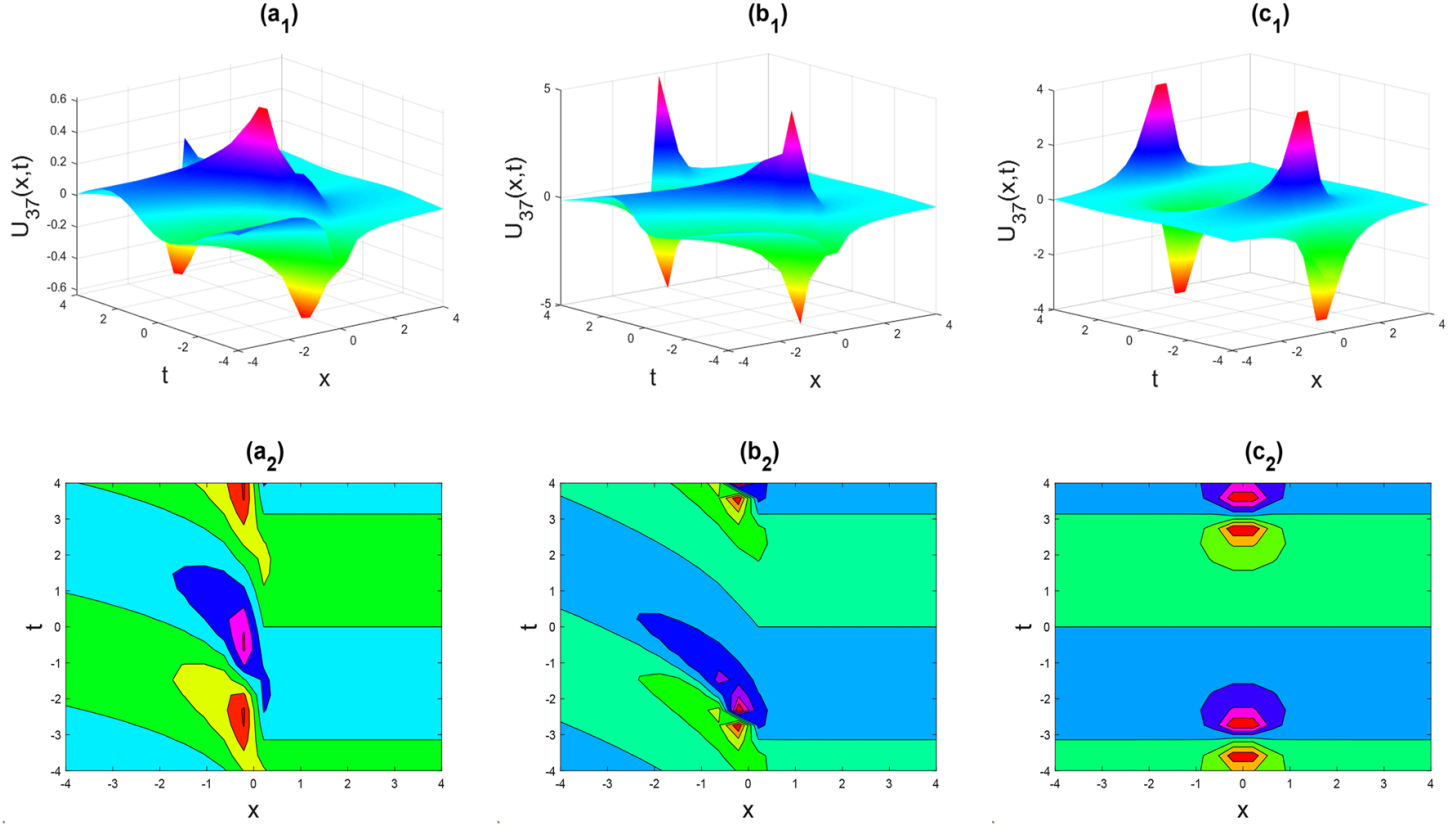
The rogue wave shape of the real part of ${U}_{37}\left(x,t\right)$ for the parameters $p=0.5, \lambda =1, \mu =1, \theta =0.3, 0.6, 1$.

**Figure 7:**
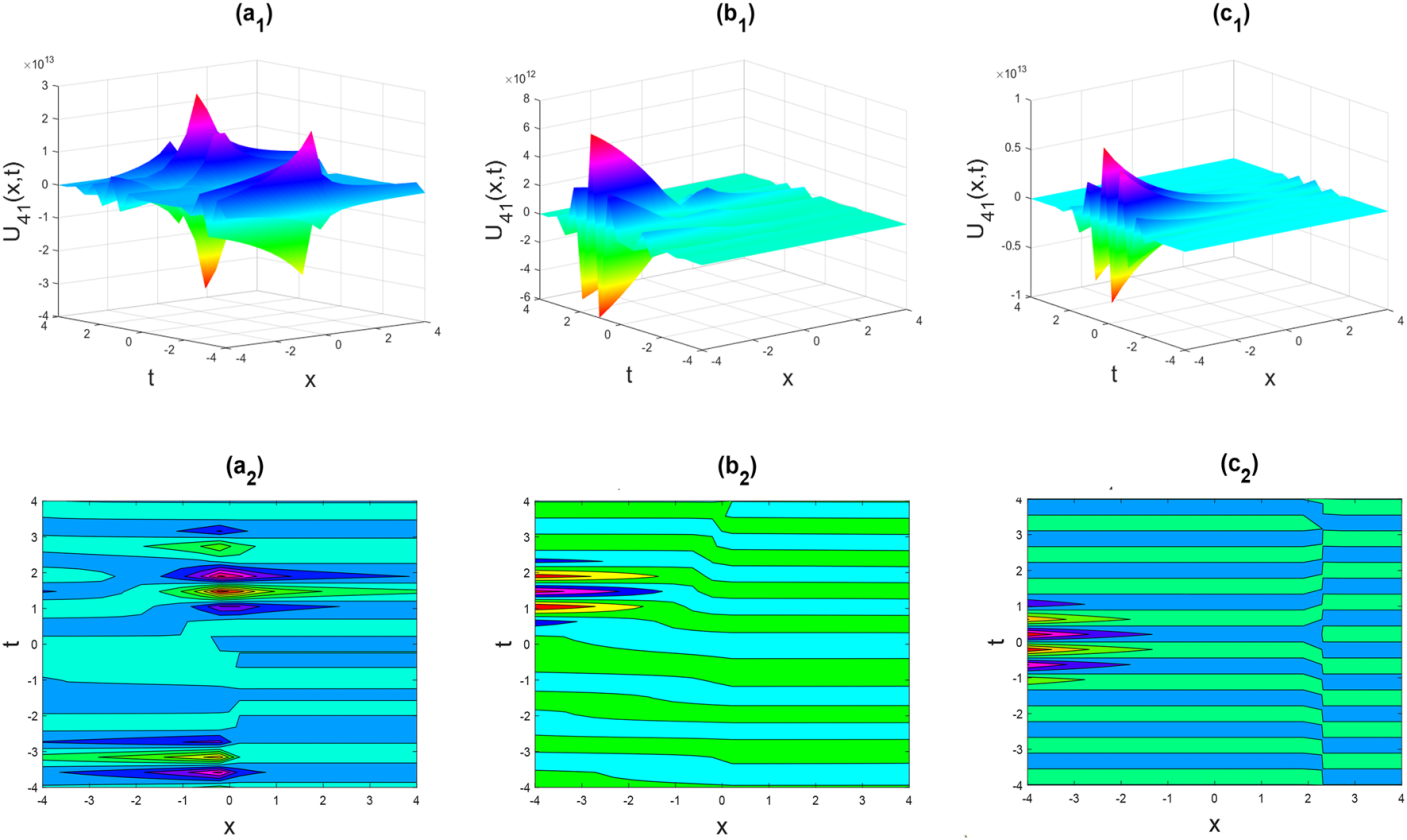
Kink shape of the imaginary part of ${U}_{41}\left(x,t\right)$ for the parameters $p=0.5, \lambda =1, \mu =1, \theta =0.3, 0.6, 1$.

**Figure 8:**
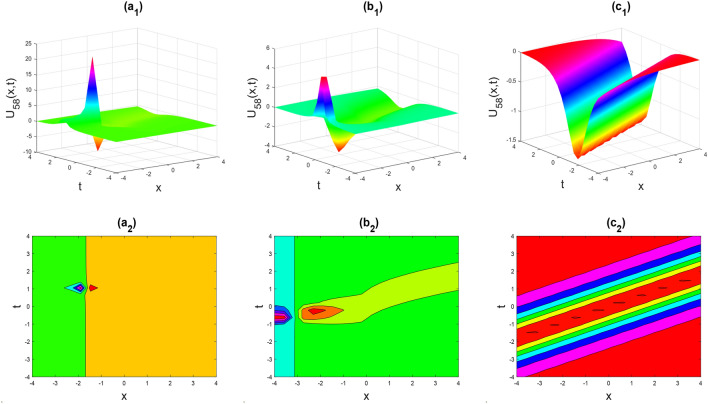
Soliton shape of the imaginary part of ${U}_{58}\left(x,t\right)$ for the parameters $p=0.5, \lambda =1, \mu =1, \theta =0.3, 0.6, 1$.

**Figure 9:**
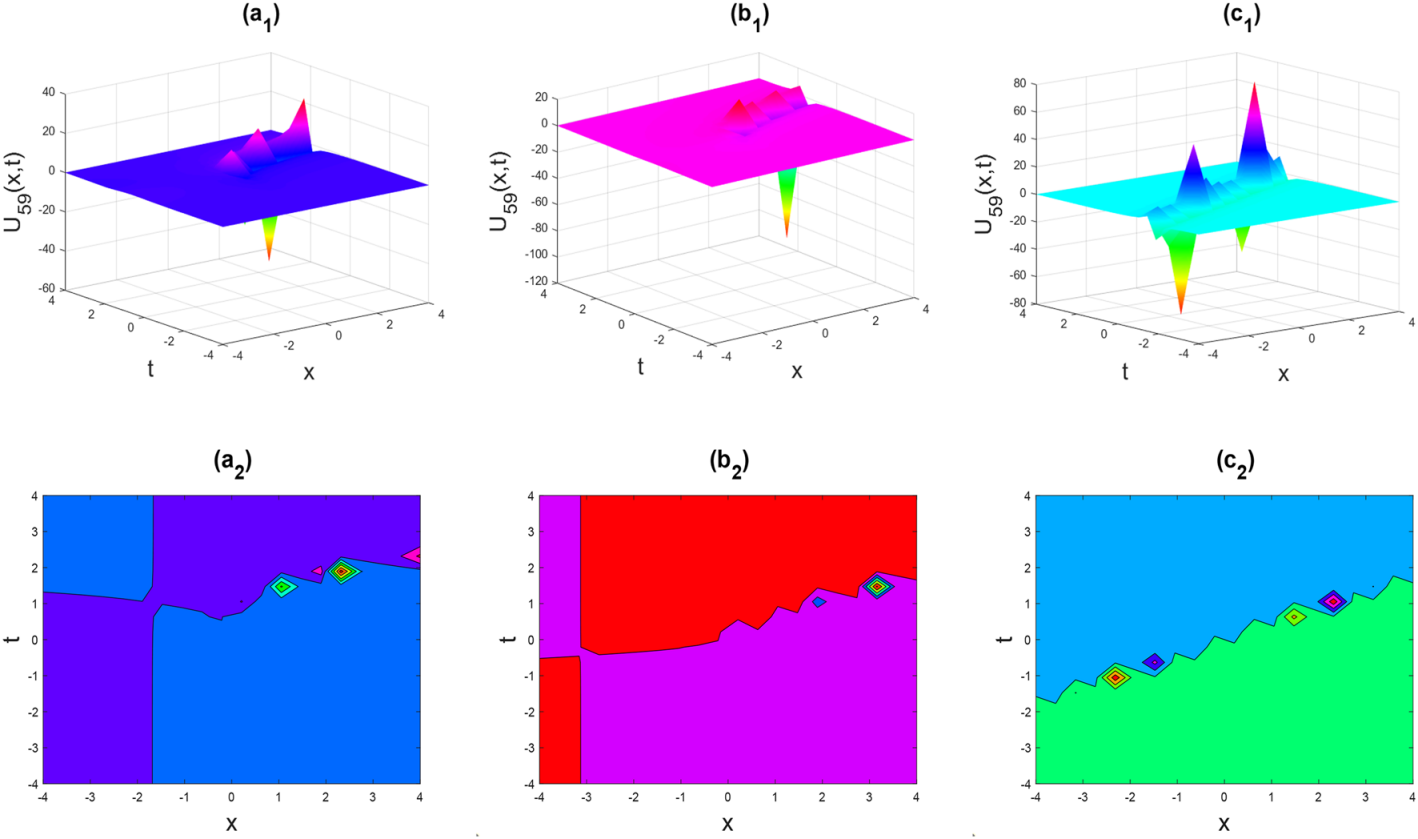
Soliton shape of the real part of ${U}_{59}\left(x,t\right)$ for the parameters $p=0.5, \lambda =1, \mu =1, \theta =0.3, 0.6, 1$.

**Figure 10:**
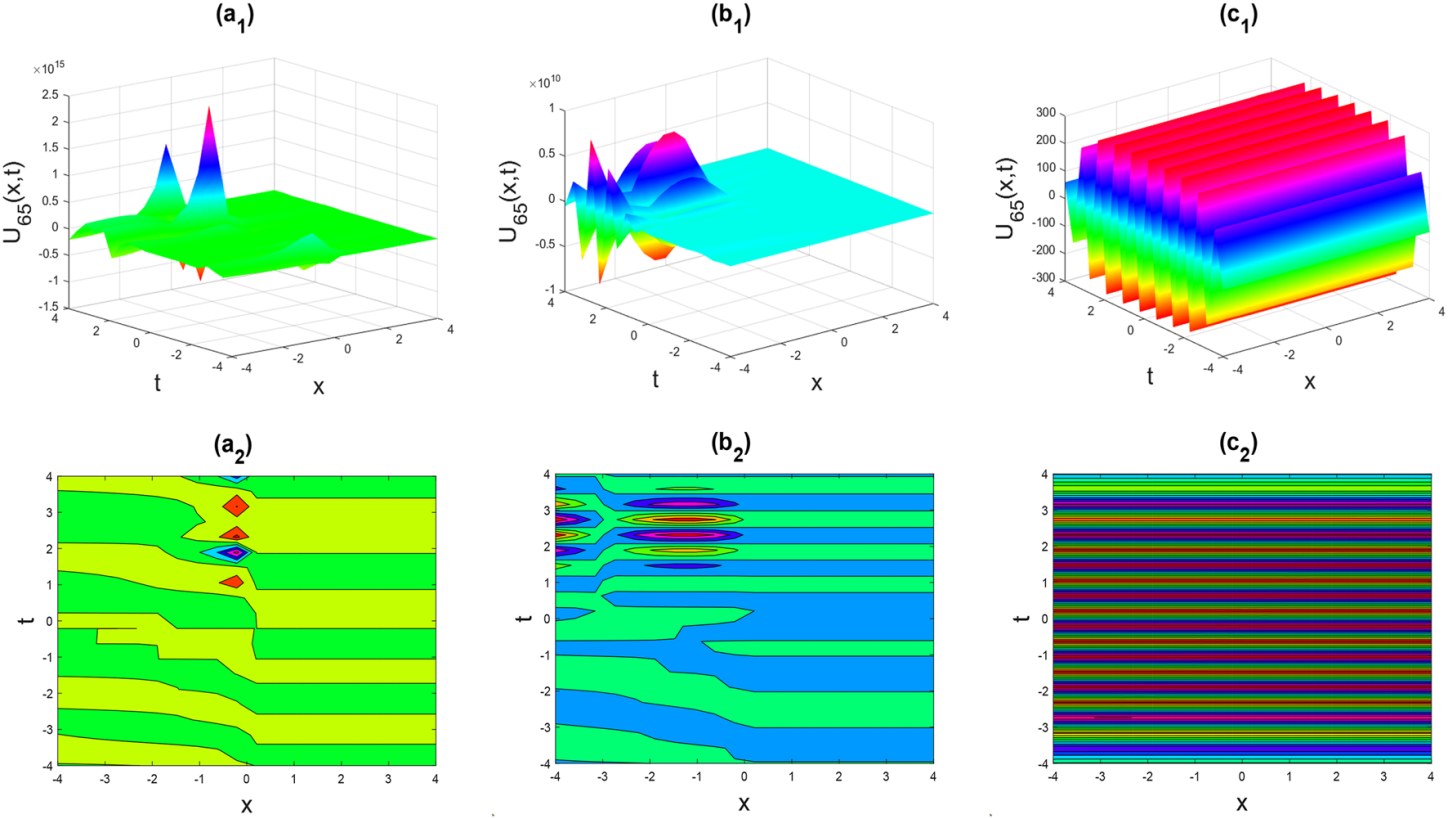
Multiple Kink-soliton shapes of the real part of ${U}_{65}\left(x,y,t\right)$ for the parameters ${a}_{0}=1, {b}_{0}=0.5, {d}_{1}=-0.1, \lambda =1, \mu =1, y=0, \theta =0.3, 0.6, 1$.

**Figure 11:**
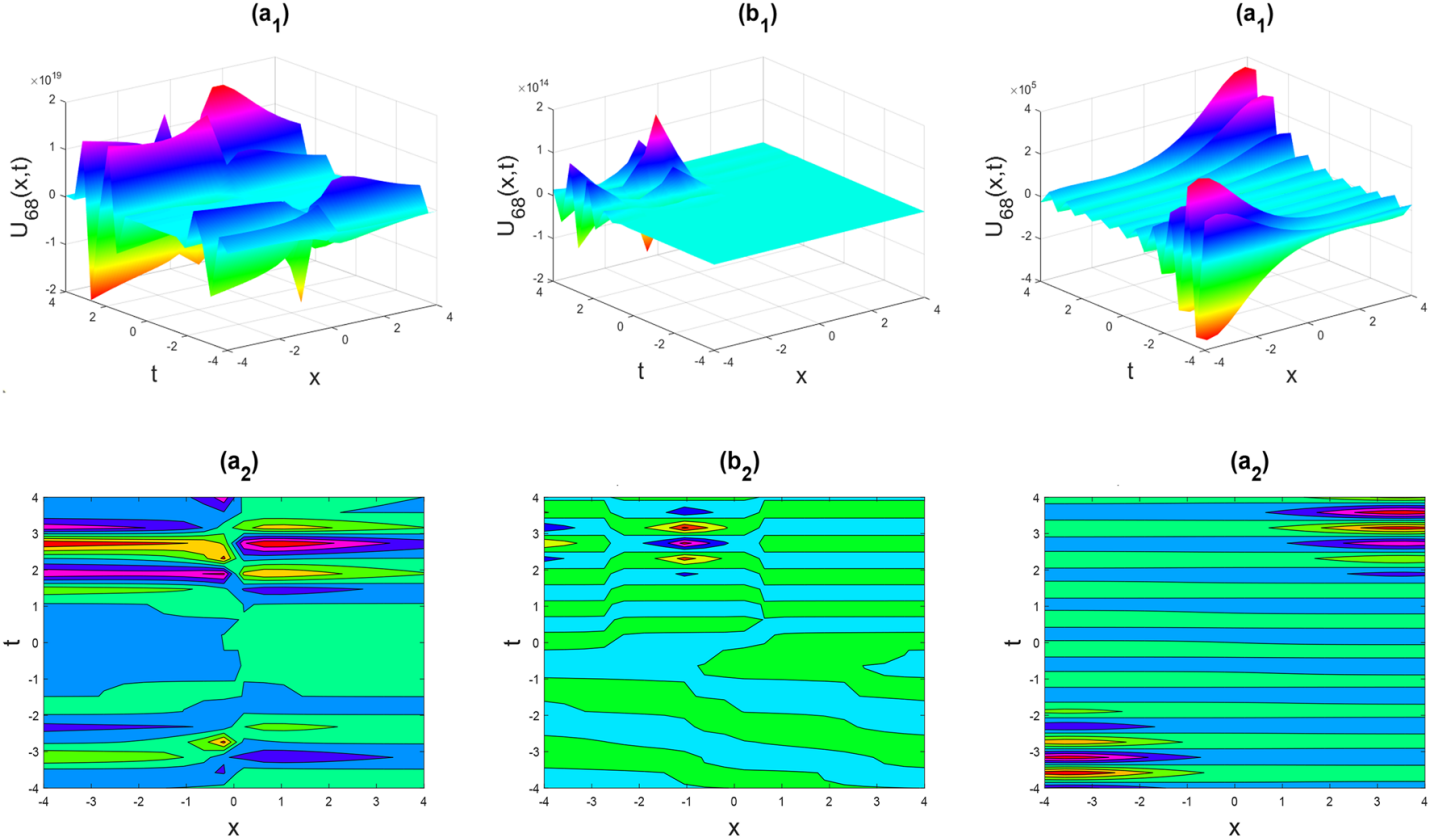
Multiple soliton shapes of the real part of ${U}_{68}\left(x,y,t\right)$ for the parameters ${a}_{0}=1, {b}_{0}=0.5, {b}_{1}=-0.1, \lambda =1, \mu =1, y=0, \theta =0.3, 0.6, 1$.

**Figure 12:**
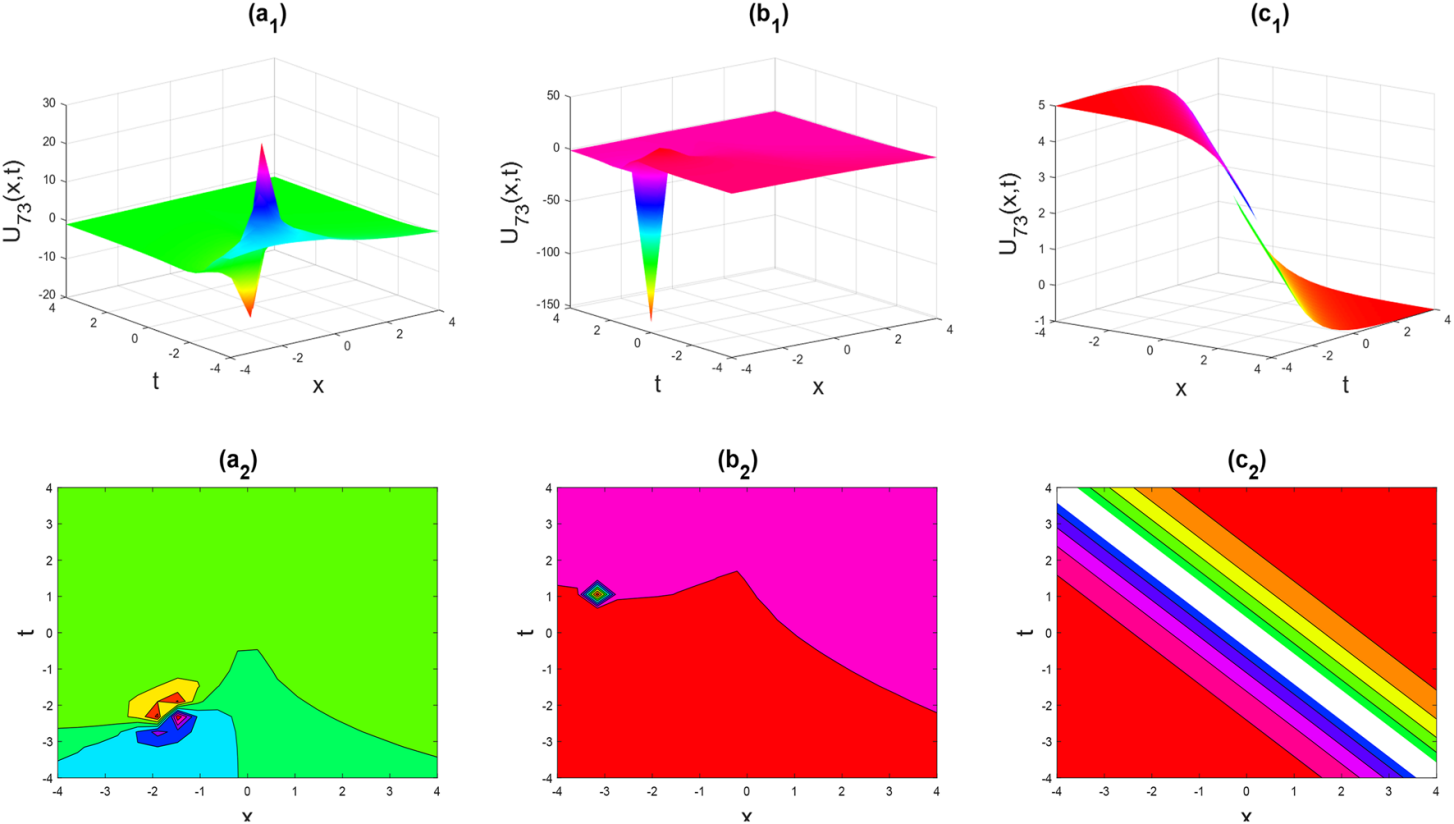
Kink-soliton shape of the real part of ${U}_{73}\left(x,y,t\right)$ for the parameters ${c}_{1}=1, {d}_{1}=0.5, \lambda =1, \mu =1, y=0, \theta =0.3, 0.6, 1$.

**Figure 13:**
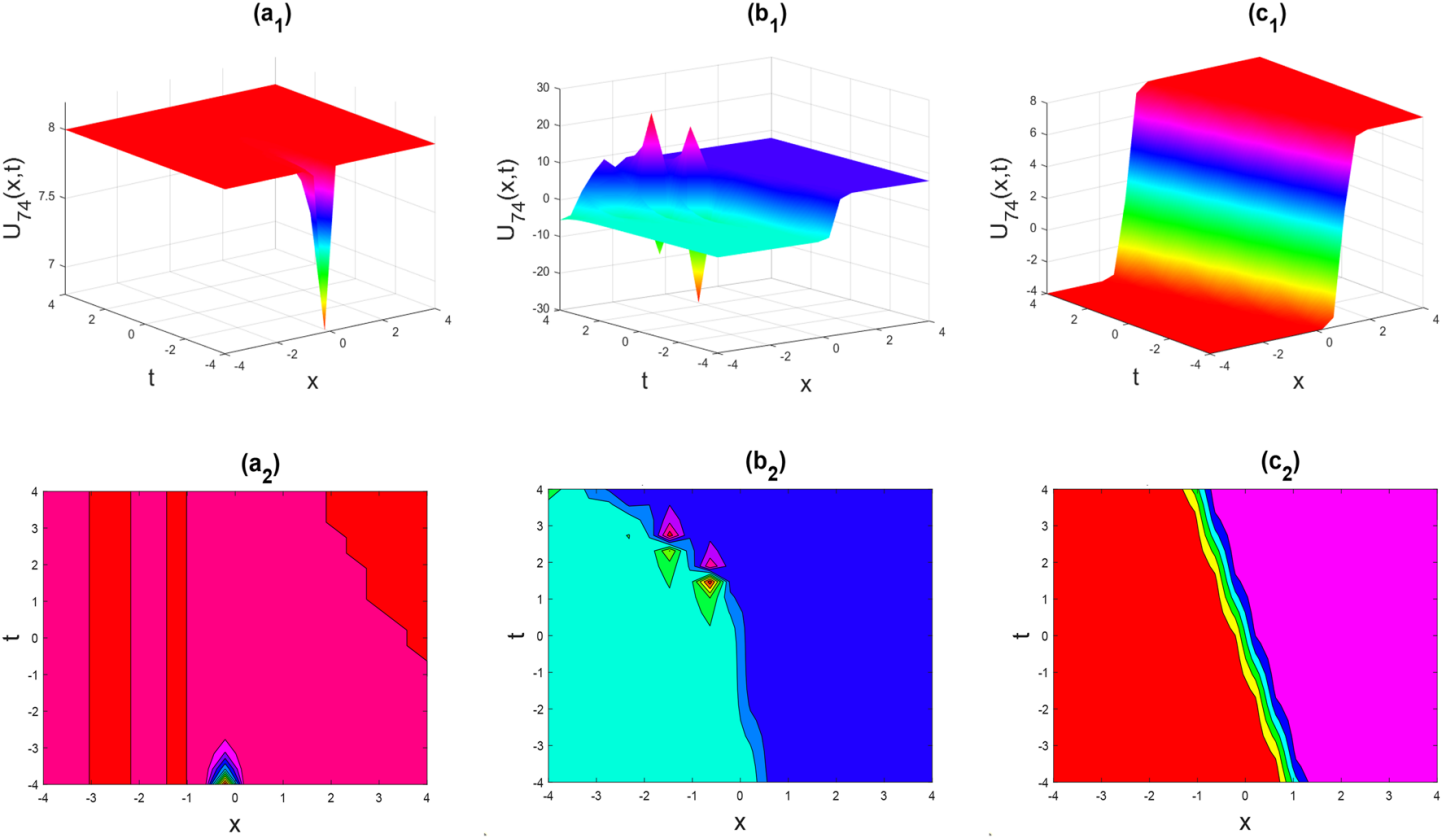
Kink-soliton shape of the real part of ${U}_{74}\left(x,y,t\right)$ for the parameters ${a}_{0}=1, {b}_{0}=0.5, \lambda =1, \mu =1, y=0, \theta =0.3, 0.6, 1$.

**Figure 14:**
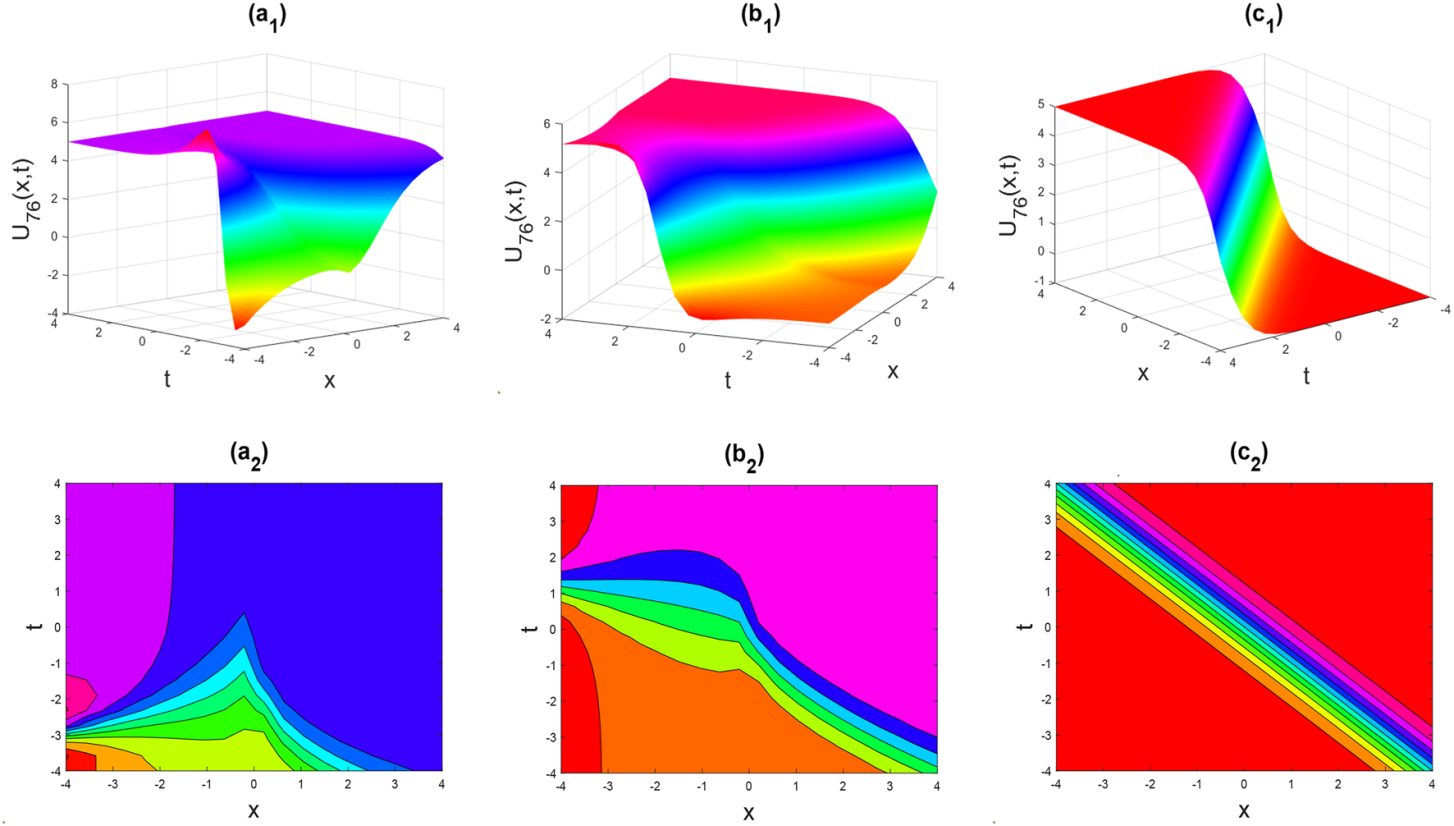
Kink shape of the real part of ${U}_{76}\left(x,y,t\right)$ for the parameters ${a}_{0}=1, {b}_{0}=0.5, \lambda =1, \mu =1, y=0, \theta =0.3, 0.6, 1$.

This section defines the recently discovered precise solutions to the time-fractional phi-four equation and the (2 + 1) dimensional CBS equation using physical and visual examples. The best way to illustrate every essential component of real-life events is through visualization. By selecting proper fractional values, we also used MATLAB's computational capabilities. We assessed its conventional features while charging various exorbitant fees for unknown factors. The detailed proofs for the equations are shown in Figs. 2, 3, 4, 5, 6, 7, 8, 9, 10, 11, 12, 13 and 14.

### Graphical and physical explanation

A famous example of this wave is a traveling wave that forms on the surface of an ocean, lake, or river. Water waves have a particular behavior because of how gravity and surface tension forces interact to determine their velocity. When the water's surface impedes, visualize a body of calm water at rest and experiencing moving waveforms. The wind blowing across the sea, dumping of an object, seismic activity, and other factors might all be factors in this disruption. Take pond ripples as an example, which are caused by the wind. Water molecules are drawn together at a body of water's surface by surface tension, which is a cohesive force. In addition, the force of gravity is pulling the water downward.

The interaction of these two forces results in a restoring force that tends to bring the water's surface back to its equilibrium position. A portion of the water molecules are forced to flow upward to form crests, which are recognized, and downward to produce troughs when the wind blows over the water, transferring energy to the surface. The disturbance propagates over the water's character due to oscillations brought on by the up-and-down motion of water molecules. When water molecules contact, they exchange energy and momentum. Gravity, surface tension, and a small amount of horizontal water molecule displacement work together to disperse these oscillations throughout the water. The wave's wavelength and the water's depth are two factors that influence how quickly a wave moves. The major influences on the wave speed of deep-water waves are the acceleration brought on by gravity and the water depth. The water wave's motion transfers energy from one place to another. The wave disperses the importance of the initial disturbance across the water's surface without displacing a large amount of water mass in the direction of propagation. Waves in water can interfere with one another and produce interference patterns. Dissipation is the term for this. Positive interference raises wave amplitudes, leading to more enormous waves than destructive interference, which can cause wave cancellation. As long-distance water waves travel through the water, friction, and viscosity eventually cause the waves to lose energy, which leads to the waves dissipating. Coastal dynamics, marine transportation, and engineering all depend on water waves. Understanding the behavior of ocean waves is essential for predicting coastal erosion, building ships, and guaranteeing maritime safety.

Figures 2, 3, 4, 5, 6, 7, 8, 9, 10, 11, 12, 13 and 14 depict the many solution versions of the time-fractional phi-four and (2 + 1) dimensional CBS equation. Each picture has two rows: the first row represents the 3D surface plot, while the second row represents the contour plot. Here, we presented the contour and 3D surface plots of many solutions. We have implemented some innovative solutions: Various shapes such as kink, multiple kink, rogue wave, soliton, lone soliton, multiple soliton, dark soliton, double soliton, and lump can be observed. The data have been altered for various values of $\theta$, where $\theta$ ranges from 0.3 to 1. When the value of $\theta$ is changed from 0.3 to 1, the solution forms can transform solitary soliton shapes to multiple soliton shapes, from singular kink-soliton shapes to multiple kink-soliton shapes, and from soliton shapes to dark soliton shapes, among others. The wave's nonlinearity induces temporal variations in its profile as it traverses the medium, but dispersion counteracts this effect, preventing the wave from diffusing or distorting. Overall, solitary waves are captivating occurrences that arise from the delicate equilibrium between dispersion and nonlinearity in a medium. These waves are crucial in several fields of physics and engineering due to their capacity to maintain their shape and propagate across long distances without dispersing or losing energy, owing to this state of equilibrium.

## Conclusion

The traveling wave solution for the time-fractional phi-four equation and the (2 + 1) dimensional CBS equation was found using the rational sine-Gordon expansion approach. A tried-and-true technique is the rational sine-Gordon expansion to resolve nonlinear partial differential equations. When the wave profile is investigated for the created generic parametric values, many depicted solitons, rogue waves, singular kink, periodic, lump, and asymptotic type solitons may be found. These solitons were constructed utilizing exponential, hyperbolic, and trigonometric structures. We have mainly focused on the influence of the values or quantities of changes for different values of one parameter ($\theta$) on the dynamic behavior of the water waves. Exponential and trigonometric functions express the calculated solutions. The space–time fractional NLS + , NLS- and UNLS models from nonlinear optics, fluid mechanics, quantum theory, and other theoretical and numerical fields will be used to explain the physical significance of the traveling wave solutions in this work. This method may be used with complex nonlinear physics, engineering, and applied mathematics models. It is conceivable that certain types of nonlinear problems may not be solvable using this methodology. While it may not be capable of handling complex nonlinear systems, it is highly efficient in solving specific types of equations. Employ other methods to verify the obtained solutions, such as asymptotic analysis, numerical simulations, or, if accessible, comparison with empirical data. This enhances the reliability and accuracy of the generated solutions. Utilise the rational Sine-Gordon expansion technique as part of a broader set of tools. Integrate additional perturbation, analytical, or numerical approaches to mitigate the limitations and enhance the method's strengths.

## Data Availability

The data used to support the findings of this study are available from the corresponding author upon request.
